# Novel prostate cancer susceptibility gene *SP6* predisposes patients to aggressive disease

**DOI:** 10.1038/s41391-021-00378-5

**Published:** 2021-05-19

**Authors:** Csilla Sipeky, Teuvo L. J. Tammela, Anssi Auvinen, Johanna Schleutker

**Affiliations:** 1 Institute of Biomedicine and FICAN West Cancer Centre, University of Turku and Turku University Hospital, Turku, Finland; 2 Department of Urology, Tampere University Hospital and Faculty of Medicine and Health Technology, Tampere University, Tampere, Finland; 3 Unit of Health Sciences, Faculty of Social Sciences, Tampere University, Tampere, Finland; 4 Department of Medical Genetics, Genomics, Laboratory Division, Turku University Hospital, Turku, Finland; 5 Present Address: UCB Pharma, Data & Translational Sciences, Braine l’Alleud, Belgium

**Keywords:** Prostate cancer, Diagnostic markers

## Abstract

Prostate cancer (PrCa) is one of the most common cancers in men, but little is known about factors affecting its clinical outcomes. Genome-wide association studies have identified more than 170 germline susceptibility loci, but most of them are not associated with aggressive disease. We performed a genome-wide analysis of 185,478 SNPs in Finnish samples (2738 cases, 2400 controls) from the international Collaborative Oncological Gene-Environment Study (iCOGS) to find underlying PrCa risk variants. We identified a total of 21 common, low-penetrance susceptibility loci, including 10 novel variants independently associated with PrCa risk. Novel risk loci were located in the 8q24 (*CASC8* rs16902147, OR 1.86, *p*_adj_ = 3.53 × 10^−8^ and rs58809953, OR 1.71, *p*_adj_ = 4.00 × 10^−6^; intergenic rs79012498, OR 1.81, *p*_adj_ = 4.26 × 10^−8^), 17q21 (*SP6* rs2074187, OR 1.66, *p*_adj_ = 3.75 × 10^−5^), 11q13 (rs12795301, OR 1.42, *p*_adj_ = 2.89 × 10^−5^) and 8p21 (rs995432, OR 1.38, *p*_adj_ = 3.00 × 10^−11^) regions. Here, we describe *SP6*, a transcription factor gene, as a new, potentially high-risk gene for PrCa. The intronic variant rs2074187 in *SP6* was associated not only with overall susceptibility to PrCa (OR 1.66) but also with a higher odds ratio for aggressive PrCa (OR 1.89) and lower odds for non-aggressive PrCa (OR 1.43). Furthermore, the new intergenic variant rs79012498 at 8q24 conferred risk for aggressive PrCa. Our findings highlighted the power of a population-stratified approach to identify novel, clinically actionable germline PrCa risk loci and strongly suggested *SP6* as a new PrCa candidate gene that may be involved in the pathogenesis of PrCa.

## Introduction

Prostate cancer (PrCa) is the second most common cancer in males and the fifth leading cause of cancer death in men worldwide (2018) [1, 2]. In Finland, it accounts for 29.6% of all newly diagnosed cancer cases and for 13.6% of all cancer deaths based on the latest NORDCAN (Cancer statistics for the Nordic countries) data (2012–2016) [3].

PrCa has a major heritable component with genetic factors accounting for 57% (95% CI 51–63%) of the variation in risk in the Nordic Twin Study of Cancer [4]. To date, genome-wide association studies (GWAS) have identified over 170 low-penetrance PrCa susceptibility loci, predominantly in populations of mixed European ancestry [5–9]. However, only a few of the identified susceptibility variants are associated with clinically relevant aggressive or advanced disease.

Previous studies have established that genetic loci, effect allele frequencies (EAF) and the strength of association (odds ratio, OR) are highly variable across geographic regions [8, 10]. In bottlenecked and isolated populations such as the Finns, many functional variants are present at relatively high frequencies because of increased drift and reduced selective pressure [11], whereas in larger outbred populations, deleterious alleles occur at low frequencies due to selection [12]. Hence, recent isolates like the Finns provide an ideal opportunity to discover disease-associated genes as underlying and initially rare variants can be encountered at higher frequencies.

In an effort to discover novel, potentially clinically actionable germline PrCa biomarkers, we conducted a genome-wide association analysis of the Finnish samples in the international Collaborative Oncological Gene-Environment Study (iCOGS).

## Materials and methods

### Study design

This study is a Finnish population-specific analysis of the single nucleotide polymorphisms (SNPs) in the iCOGS genome-wide custom genotyping array [5] for overall PrCa risk and subsequent testing for aggressive disease. The iCOGS study was designed by the international consortium to detect genetic variants related to prostate, breast and ovarian cancers [13]. The study protocol was approved as described in the iCOGS study [5].

### Study participants

After application of quality control criteria, the final analysis was based on 2738 PrCa cases and 2400 controls without a known diagnosis of PrCa. Of the cases, 2283 were clinically diagnosed patients from the Pirkanmaa Hospital District confirmed from medical records. Another set of patients consisted of 455 cases of the Finnish Randomized Study of Screening for Prostate Cancer (FinRSPC) [14, 15]. The FinRSPC trial population and the study protocol have been described in detail elsewhere [16]. Cancer free control subjects (*n* = 2.400) were identified through the FinRSPC trial [14]. All of the samples were collected with written and signed informed consent. The cancer diagnoses were confirmed using medical records and the Finnish Cancer Registry. The study was approved by the research Ethics committee at Pirkanmaa Hospital District (tracking numbers R10167, 90577, R03203) and by the National Supervisory Authority for Welfare and Health (VALVIRA).

Clinical characteristics of the genotyped PrCa patients are summarised in Table 1. PSA at diagnosis was classified as ≤20 versus >20 ng/mL. Gleason score was divided into ≤6, 7 and ≥8. Stage was divided into organ-confined (T1-2, N0/x, M0/x) versus advanced disease (T3–4, or N1 or M1). PrCa death was defined based on the underlying cause recorded as the official cause of death by Statistics Finland. Aggressive PrCa was defined as having PSA at diagnosis >20 ng/mL, or Gleason Score ≥8, or T3/T4, or N1, or M1, or PrCa-specific mortality (PCM). Comprehensive definition of non-aggressive PrCa was the following: PSA at diagnosis ≤20 ng/mL, and Gleason Score ≤6, and not T3/T4, and not N1, and not M1, and not PCM.

**Table 1:** Clinical characteristics of Finnish prostate cancer patients.

Total PrCa sample size	*n* = 2738 (%)
Age at diagnosis (years)	
≤55, young onset	106 (3.90)
>55	2632 (96.1)
Diagnostic PSA level, ng/mL	
Low, ≤20	2099 (76.7)
High, >20	484 (17.3)
Missing data	155 (5.66)
Gleason score	
Low, ≤6	1320 (48.2)
High, ≥8	368 (13.4)
Gleason 7	685 (25.0)
Missing data	365 (13.3)
T stage	
T0/Tx	13 (0.48)
T1	1105 (40.4)
T2	972 (35.5)
T3	443 (16.2)
T4	97 (3.54)
Missing data	108 (3.95)
N stage	
N0/Nx	2616 (95.5)
N1	14 (0.51)
Missing data	108 (3.95)
M stage	
M0/Mx	2439 (89.1)
M1	191 (6.98)
Missing data	108 (3.95)
PSA progression	
Progressed	960 (35.1)
Missing data	1778 (65.0)
Vital status	
Deceased of PrCa	298 (10.9)
Deceased of else	928 (33.9)
Alive	1512 (55.2)
Aggressive PC	
Yes^a^	1019 (55.9)
No^b^	804 (44.1)

^a^Aggressive prostate cancer is defined as PSA at diagnosis >20 ng/mL or Gleason Score ≥8 or T3/T4 or N1 or M1 or PCM.

^b^Non-aggressive prostate cancer is defined as PSA at diagnosis ≤20 ng/mL and Gleason Score ≤6 and not T3/T4 and not N1 and not M1 and not PC.

### Genotype quality control

Altogether, *n* = 211,155 SNPs were genotyped in iCOGS for Finnish subjects. Systematic quality control (QC) steps were conducted on the raw iCOGS genotyping data. Females, individuals with low call rate, individuals with extreme heterozygosity, known or cryptic duplicates, individuals not matching previous genotyping and ethnic outliers have been left out. Subsequently, first-degree relatives, duplicate subjects, and cases missing clinical data have been removed as well. The exclusion criteria for SNPs were a genotyping call rate less than 95%, failing the missingness test (GENO > 1, default ---geno value of 0.0 was used), minor allele frequency (MAF < 1 × 10^−6^ or > 0.499) and genotype frequency that deviated from expected Hardy–Weinberg equilibrium among control samples (*P* ≤ 0.05). After frequency and genotype pruning, 185,478 SNPs were retained for analysis [5].

### Statistical analyses

Standard procedures for case-control GWAS were executed [17, 18]. The association between each SNP and PrCa was estimated by per-allele OR and 95% CI using unconditional logistic regression implemented in PLINK (v1.07) [19] assuming an additive genetic model. We used a *p* value threshold of 5.0 × 10^−8^ to determine genome-wide significance. False discovery rate (FDR)-adjusted significance was set to *p*_adj_ < 4 × 10^−5^ using the Benjamini–Hochberg method. The EAF was set to >5%. The Hardy–Weinberg equilibrium equation was used to determine whether the proportion of each genotype obtained was in agreement with the expected values as calculated from the allele frequencies.

Identified PrCa susceptibility variants were pruned by pairwise threshold, removing loci with a high level of linkage disequilibrium (LD) (*r*^2^ > 0.5), resulting in independent signals for PrCa risk (PLINK v1.07) [19]. The variants were then tested in a case-control setting for the risk of aggressive PrCa defined by a Gleason Score ≥8 and for the risk of non-aggressive PrCa defined by a Gleason Score ≤6. In addition, we assessed the association of the genetic variants with the comprehensively defined entity of aggressive PrCa and with the comprehensively defined non-aggressive PrCa (for definition see above) (IBM^®^ SPSS^®^ Statistics Version26 for Mac SPSS Inc., Chicago, IL, USA).

### Annotation

Ensembl was used for gene annotation [20] indicating HGNC gene symbols from the HUGO Gene Nomenclature Committee [21]. Variant annotation and functional effect prediction was performed with the Variant Effect Predictor (VEP) [22] and SnpEff [23].

## Results

### Prostate cancer susceptibility

Altogether, we identified 160 PrCa susceptibility loci at GWAS significance (*p* < 5 × 10^−8^, *p*_adj_ < 4 × 10^−5^, Supplementary Table 1 and Supplementary Fig. 1). After genotype pruning, 21 common, low-penetrance susceptibility loci were independently associated with PrCa risk with per-allele ORs ranging between 1.86 and 0.74 (Table 2.). Association of the 10 novel variants with malignant neoplasm of prostate has been validated using the FinnGen and UKBB biobank data (Supplementary Table 2, http://r3.finngen.fi)

**Table 2:** Summary results for 21 loci independently associated with prostate cancer risk.

Marker	Locus	Position^a^	Alleles^b^	EAF case	EAF control	OR^c^ (95% CI)	*P* value	*P*_adj_value^d^	Nearby genes
**rs16902147**	**8q24**	**128474254**	**GA**	**0.07**	**0.04**	**1.86 (1.56**–**2.23)**	**1.214E**−**11**	**3.525E**−**8**	**CASC8**
**rs79012498**^e^	**8q24**	**128222502**	**GA**	**0.08**	**0.04**	**1.81 (1.53**–**2.15)**	**1.507E**−**11**	**4.26E**−**8**	**PRNCR1-CASC19 (intergenic)**
rs11650494	17q21	44700185	AG	0.06	0.04	1.76 (1.46–2.12)	2.817E−9	3.981E−6	RP1-62O9.3-ZNF652 (intergenic)
**rs58809953**	**8q24**	**128477243**	**AG**	**0.07**	**0.04**	**1.71 (1.43**–**2.04)**	**2.855E**−**9**	**4.001E**−**6**	**CASC8**
**rs2074187**^e^	**17q21**	**43285358**	**AC**	**0.07**	**0.04**	**1.66 (1.38**–**1.98)**	**3.743E**−**8**	**3.752E**−**5**	**SP6**
rs9656816	8q24	128603836	GA	0.17	0.12	1.43 (1.28–1.60)	2.247E−10	5.165E−7	CASC8-CASC11 (intergenic)
**rs12795301**	**11q13**	**68748861**	**AC**	**0.13**	**0.098**	**1.42 (1.25**–**1.61)**	**2.786E**−**8**	**2.894E**−**5**	**RP11-554A11.8-MYEOV (intergenic)**
**rs995432**	**8p21**	**23578796**	**GA**	**0.53**	**0.45**	**1.38 (1.28**–**1.50)**	**7.575E**−**16**	**3.003E**−**11**	**RP11-583M2.2-NKX3-1 (intergenic)**
rs4871798	8q24	128549145	AG	0.28	0.23	1.33 (1.22–1.46)	3.105E−10	6.716E−7	CASC8
rs6985504	8q24	128565958	AG	0.35	0.30	1.27 (1.17–1.38)	1.482E−8	1.649E−5	CASC8
rs1447293	8q24	128541502	GA	0.50	0.44	1.26 (1.16–1.36)	5.639E−9	7.596E−6	CASC8
rs4793976	17q21	44135496	GA	0.39	0.33	1.26 (1.17–1.37)	1.216E−8	1.397E−5	LINC02086
rs3760511	17q12	33180426	CA	0.49	0.43	1.26 (1.16–1.36)	7.107E−9	9.01E−6	HNF1B
rs4793529	17q24	66630231	GA	0.47	0.53	0.80 (0.74–0.87)	2.416E−8	2.557E−5	CASC17
**rs4871790**	**8q24**	**128510716**	**CA**	**0.45**	**0.50**	**0.80 (0.74**–**0.87)**	**1.658E**−**8**	**1.809E**−**5**	**CASC8**
**rs587948**	**8q24**	**128410862**	**CA**	**0.34**	**0.39**	**0.80 (0.74**–**0.87)**	**2.819E**−**8**	**2.894E**−**5**	**CASC8**
**rs2739459**	**19q13**	**56060886**	**GA**	**0.42**	**0.48**	**0.79 (0.73**–**0.86)**	**2.186E**−**8**	**2.355E**−**5**	**KLK2**
**rs757138**	**7p15**	**27955927**	**CA**	**0.26**	**0.31**	**0.78 (0.72**–**0.85)**	**1.114E**−**8**	**1.289E**−**5**	**JAZF1**
rs266876	19q13	56052629	GA	0.19	0.23	0.76 (0.69–0.84)	2.745E−8	2.868E−5	KLK3
rs10505477	8q24	128476625	GA	0.46	0.53	0.74 (0.69–0.80)	6.105E−14	4.785E−10	CASC8
rs2659051	19q13	56037380	GC	0.12	0.16	0.72 (0.64–0.80)	5.894E−9	7.847E−6	AC011523.2

^a^Position is based on Human Genome version 37p13 (hg19).

^b^Effect allele/Other allele.

^c^Per-allele odds ratio for the effect allele.

^d^Adjusted for false discovery rate (FDR) using the Benjamini–Hochberg method bold, newly reported for prostate cancer risk grey highlighted, variants associated with aggressive prostate cancer.

^e^Markers associate with aggressive prostate cancer.

In this study, the EAFs of common PrCa susceptibility variants ranged between 0.06 and 0.53. The identified PrCa risk loci spanned nine different gene regions altogether with five of the associated loci being intergenic. Most of the PrCa susceptibility variants were detected in the *CASC8* gene (*n* = 8), whereas *SP6, CASC17, JAZF1, HNF1B, KLK2, KLK3, AC011523.2*, and *LINC02086* possessed a single variant each.

Based on functional annotation of the identified PrCa-associated variants, intronic variants were most frequent (10 SNPs, 48%), followed by intergenic variants (5 SNPs, 24%), and there were equal numbers of upstream and downstream intronic gene variants (both 3 SNPs, 14%). These findings highlight the possible importance of transcriptional regulation in PrCa.

The identified 13 risk signals were condensed at chromosomal regions 8q24, 17q21, 11q13, 8p21, and 17q12 (OR 1.86–1.26), whereas the eight protective variants were situated at 19q13, 8q24, 7p15, and 17q24 (OR 0.72–0.80). Chromosomes 11 and 17 appeared to be exclusively risk-conferring, whereas chromosomes 7 and 19 possessed solely protective variants. Exclusively risk genes identified in this study were predominantly transcription factors (*SP6, HNF1B*, *LINC02086*). On the other hand, SNPs in *CASC17*, *KLK2*, *KLK3, JAZF1*, and *AC011523.2* were solely protective.

The strongest risk effect was found for the novel intronic variant rs16902147 in the *CASC8* (cancer susceptibility candidate 8) gene at 8q24 with an OR of 1.86 (95% CI 1.56–2.23; *p*_adj_ = 3.53 × 10^−8^) and EAF of 0.07. The statistically most significant signal originated from the intergenic variant (RP11-583M2.2-NKX3-1) rs995432 at 8p21 (*p*_adj_ = 3.00 × 10^−11^). This finding confirmed the previous GWAS findings at these genomic locations [24, 25] and strengthened these observations with the new variants.

Out of the 21 identified PrCa susceptibility hits, 10 (48%) were novel variants not reported earlier in association with PrCa susceptibility. Novel loci with high effect sizes were located in 8q24 (*CASC8* rs16902147, OR 1.86, *p*_adj_ = 3.53 × 10^−8^ and rs58809953, OR 1.71, *p*_adj_ = 4.00 × 10^−6^; intergenic rs79012498, OR 1.81, *p*_adj_ = 4.26 × 10^−8^) and 17q21 (*SP6* rs2074187, OR 1.66, *p*_adj_ = 3.75 × 10^−5^) regions and had low EAF (≤0.08). Additionally, two novel intergenic variants, rs12795301 at 11q13 (OR 1.42, *p*_adj_ = 2.89 × 10^−5^) and rs995432 at 8p21 (OR 1.38, *p*_adj_ = 3.00 × 10^−11^), showed risk for overall PrCa. Novel protective variants were located in *CASC8* (rs4871790 and rs587948, for both OR 0.80), *KLK2* (rs2739459, OR 0.79) and *JAZF1* (rs757138, OR 0.78) genes. Interestingly, they showed relatively high EAFs of 0.26–0.45. The most important finding was a possible new PrCa risk gene, *SP6*, that had not yet been implicated as a potential causal gene for PrCa.

### Aggressive prostate cancer susceptibility

To explore whether the identified PrCa susceptibility loci were associated with aggressive disease, we analysed their association with a high Gleason Score ≥8 and a low Gleason Score ≤6 and with comprehensively defined aggressive PrCa and non-aggressive PrCa (see Methods). Findings are summarised in Table 3. The intronic variant rs2074187 in *SP6* was associated with higher OR for high Gleason score disease (OR 2.09, *p* = 0.000005) than for low Gleason score disease (OR 1.50, *p* = 0.0004) or overall PrCa (OR 1.66, *p* = 3.752 × 10^−5^). Similarly, it was associated with a higher effect size for comprehensively defined aggressive PrCa (OR 1.89, *p* = 4.738 × 10^−8^) than non-aggressive PrCa (OR 1.43, *p* = 0.008) or overall PrCa (OR 1.66, *p* = 3.752 × 10^−5^). Furthermore, we revealed an association between the new intergenic variant rs79012498 at 8q24 (*PRNCR1-CASC19*) and aggressive PrCa. The ORs for high Gleason score and aggressive PrCa (OR 2.14 and OR 2.10, respectively) were higher than for low Gleason score and non-aggressive PrCa (OR 1.76 and OR 1.57, respectively), or for overall PrCa (OR 1.81).

**Table 3:** Association of novel variant in *SP6* and in 8q24 intergenic regions with risk of aggressive prostate cancer, non-aggressive prostate cancer and overall prostate cancer.

Marker	Locus/Nearest gene	EAF Controls	Overall prostate cancer	High Gleason score disease (≥8)	Low Gleason score disease (≤6)	Aggressive^a^	Non-aggressive^b^
			OR (95% CI, *p*), EAF				
		*n* = 2400	*n* = 2738	*n* = 368	*n* = 1320	*n* = 1019	*n* = 804
rs2074187 A/C^c^	17q21/SP6	0.042	1.66 (1.38–1.98, 3.752E−5), 0.065	**2.09** (1.53–2.87, 0.000005), 0.082	1.50 (1.20–1.87, 0.0004), 0.060	**1.89** (1.50–2.37, 4.738E−8), 0.074	1.43 (1.10–1.86, 0.008), 0.058
rs79012498 G/A^c^	8q24/PRNCR1-CASC19	0.044	1.81 (1.53–2.15, 4.26E−8), 0.076	**2.14** (1.57–2.91, 0.000001), 0.084	1.76 (1.43–2.17, 1.215E−7), 0.073	**2.10** (1.69–2.61, 2.851E−11), 0.084	1.57 (1.22–2.01, 0.0005), 0.065

Bold entries show most significant ORs with aggressive clinical variables.

Case-control analyses.

^a^Aggressive prostate cancer is defined as PSA at diagnosis >20 ng/mL or Gleason Score ≥8 or T3/T4 or N1 or M1 or PCM.

^b^Non-aggressive prostate cancer is defined as PSA at diagnosis ≤20 ng/mL and Gleason Score ≤6 and not T3/T4 and not N1 and not M1 and not PCM.

^c^Effect allele/Other allele.

The EAF for both the *SP6* rs2074187 and the intergenic rs79012498 variant was clearly higher in aggressive PrCa compared to non-aggressive PrCa (*p* ≤ 0.05) or in controls (*p* < 0.00001).

## Discussion and conclusions

This population-specific GWAS addressed the major challenge of the basis of inheritance of PrCa by discovering germline biomarkers for aggressive disease in the Finnish population. We identified 21 independent PrCa susceptibility loci demonstrating statistically significant association after FDR correction, including 10 novel germline variants. In addition, we not only proposed *SP6* as a new PrCa risk gene that had not yet been implicated as a potential causal gene for PrCa, but we also linked the *SP6* rs2074187 intronic variant to aggressive disease outcomes. Furthermore, we showed a new intergenic variant (rs79012498) at 8q24 *PRNCR1-CASC19* conferred risk of aggressive PrCa.

The vast majority of the 21 identified PrCa susceptibility variants were intronic in this study. Non-coding variants were reported to play a role in distinguishing PrCa, metastatic PrCa, and castration-resistant metastatic PrCa [26] and could pave the way for identifying novel treatment paradigms [27]. Mechanistic explanations for the effect of some non-coding variants do exist. For example, the rs11672691 SNP at 19q13 was associated with aggressive PrCa and creates a transcription factor binding site that in turn promotes oncogenesis by impacting expression of nearby genes [28].

Previous studies have demonstrated the utility of bottleneck populations to enable the discovery of rare but high-impact, disease-associated variants due to their enrichment in these populations [29–31]. Our study suggests a similar phenomenon with the 10 newly identified PrCa susceptibility loci. Interestingly, the EAF of the new risk variants was rather low (EAF 0.07–0.013), which might be the result of genetic drift [11]. Except for the rs995432 SNP at 8p21 (EAF 0.53). In contrast, the EAF of the new protective variants are condensed at high levels (EAF 0.26–0.45), and the EAFs of earlier reported PrCa risk alleles are uniformly distributed [6, 7].

The direction and strength of the associations of the PrCa-related variants often differ across populations. The per-allele OR of the new PrCa risk variants found in this study was in the higher range (OR 1.86–1.38) of previously identified, common, low-penetrance PrCa susceptibility loci as reviewed [5–7, 32], where each variant individually modestly modified the risk of PrCa. Similarly, the protective variants described here (OR 0.78–0.80) were more protective than the earlier established SNPs [5–7, 32].

To date, one of the strongest PrCa risk factors is the newly identified rs16902147 and rs58809953 in *CASC8*. *CASC8* is a long non-coding RNA (lncRNA) gene located in the gene desert region of 8q24 near the *MYC* gene [33]. *CASC8* gene itself has been implicated in PrCa risk [34] as its variants could potentially affect transcription factor binding [33]. The 8q24 region is a known PrCa susceptibility hot-spot, harbouring multiple risk variants where lncRNAs have been implicated [35]. Our findings support earlier observations that lncRNAs at 8q24 play a key role in PrCa aetiology [36–38].

The three newly identified intergenic risk variants were located in 11q13, 8p21 and 8q24. The 11q13 region has been previously linked to PrCa risk, where a rare intronic variant (IVS6-43A > G) in the *EMSY* gene has been associated with aggressive unselected PrCa cases [39]. Nurminen et al. found two more independent regions at 11q13 associated with PrCa risk (rs10899221 in *EMSY*, rs12277366 intergenic) [40]. Previous research has pointed to the 8p21 region [41] where frequent alteration in the prostate oncogenome has been associated with loss of androgen-regulated prostate-specific *NKX3.1* homeobox transcription factor gene [42].

The rs79012498 novel intergenic variant at 8q24 was associated with aggressive PrCa in this study. It lies at the hypothetical locus *LOC105375752* of a lncRNA gene between *PRNCR1* and *CASC19*. The *LOC105375752* locus itself has been reported to be a PrCa GWAS locus [43, 44] but has not been associated with aggressive PrCa. *PRNCR1* (*PCAT8*) is similarly a lncRNA and reported PrCa risk locus [44]. *PRNCR1* is highly overexpressed in aggressive PrCa [45]. *PRNCR1*, together with *PCGEM1*, bind to an androgen receptor (*AR*) and strongly enhance androgen-receptor-mediated gene activation programmes and proliferation in PrCa cells, thereby circumventing androgen-deprivation therapy [45]. *PRNCR1* is upregulated in PrCa and prostatic intraepithelial neoplasia cells and attenuates cell viability and activity of the *AR* when knocked down [46]. The other nearest gene to the rs79012498 variant is *CASC19* (cancer susceptibility 19), which is likewise a tumour risk lncRNA gene [44]. A rare segregating haplotype, including *PRNCR1* and *CASC19* gene variants in the region of 8q24, has been identified in familial PrCa samples as a cancer predisposition locus [37].

The newly identified *SP6* candidate gene for PrCa is a transcription factor gene [47]. Transcription factors are cellular proteins, and by regulating the transcription of genes they offer promising therapeutic targets for RNA interference therapy in PrCa [48]. The *SP6* gene, also known as *EPFN* or *KLF14* or *EPIPROFIN*, encodes an intracellular transcription factor protein. It belongs to a family of transcription factors that contain 3 classical zinc finger DNA-binding domains consisting of a zinc atom tetrahedrally coordinated by 2 cysteines and 2 histidines (C2H2 motif). These transcription factors bind to GC-rich sequences and related GT and CACCC boxes [49]. Interestingly, *SP6* RNA expression is enhanced in ductus deferens, seminal vesicles and placenta, but not in prostate [50]. Predicted localisation is intracellular and, mainly in the nucleoplasm. *SP6* has two transcripts and different splice variants. Variant rs2074187 in *SP6* was associated with aggressive PrCa risk and suggestively shows potential as a novel germline genetic marker. This SNP encodes transcript variant 1, which represents the longer transcript of the gene [51]. The higher effect size of rs2074187, differentiating aggressive PrCa (OR 1.89) from non-aggressive disease (OR 1.43), is remarkable compared to previously identified aggressive loci (OR 1.12–2.3) [52–54], and Supplementary Table 3. The EAF of 0.07 in Finnish cases in our discovery cohort is comparable with EAFs of earlier identified aggressive PrCa risk loci [53–55].

Interestingly, the *SP6* transcription factor gene is located in 17q21, which is close to *HOXB13*. The G84E mutation of *HOXB13* has been linked to significantly increased PrCa risk [56, 57], especially in Finns. Previously, we showed a synergistic effect between *HOXB13* (G84E) and *CIP2A* (R229Q) strongly predisposing patients to aggressive PrCa [55]. However, the *HOXB13* G84E risk variant only partially explained the linkage signal to 17q21 observed in Finns earlier [58]. Our finding of *SP6* as a new, potential PrCa risk gene may explain the remaining part of this linkage, which warrants follow-up.

*SP6* was previously associated with β-catenin-mediated prostate tumourigenesis [59]. The confounding role of androgen signalling in β-catenin-mediated oncogenic transformation in prostate tumourigenesis has been shown through upregulation of the *SP6* gene among others in microarray analyses of transcriptional profiles in mice [59].

*SP6* has also been implicated in breast cancer therapy resistance and linked to the regulation of the Wnt-BMP signalling pathway [60]. An important paralog of the *SP6* protein coding gene is *SP8*, which was previously identified as a candidate gene (rs12155172, *p* = 4.95 × 10^−13^) associated with PrCa susceptibility in European ancestry samples [5].

Like the *SP6* gene, many of the previously identified PrCa genes are transcription factors (e.g., *HOXB13, AR, HNF1B, FOXA1, NKX3.1*), and their binding is often affected by sequence variations [61]. DNA transcription-related genes have been justified as the largest molecular functional group in gene set enrichment analyses [62]. This finding may point to the possible implications of RNA interference therapy in the future [48].

In summary, we report a new PrCa risk gene, *SP6*, that is also associated with aggressive disease outcomes. Findings in this study demonstrate the utility of population-specific approach and the power of homogenous populations to discover disease-specific SNPs that have not been revealed in mixed European studies.

At the same time, homogeneous population material provided a resource to validate previous findings from mixed European populations shown by finding a number of previously identified, important PrCa susceptibility genes (*CASC8, HNF1B, JAZF1, CASC17, KLK2, KLK3*).

This population-specific approach is further strengthened and justified by the FinnGen study identifying top hits for malignant neoplasma of prostate, e.g. *POU5F1B, HOXB13, HOXB7, SKAP, NPEPPS, GNGT2* (http://r3.finngen.fi/top_hits).

Consequently, this study reports a novel gene and candidate variants for investigation of the pathogenesis of PrCa. Variants presented in this study are optimal candidates for functional studies to further investigate the molecular mechanisms and biological effects underlying this association and the role of the 17q21 and 8q24 regions in PrCa development.

## Supplementary information

Supplementary material
