# Supplementary material for: Novel prostate cancer susceptibility gene SP6 predisposes patients to aggressive disease
Source: Prostate Cancer Prostatic Dis. 2021 May 19;24(4):1158–66. doi: 10.1038/s41391-021-00378-5 (PMC8616752; doi:10.1038/s41391-021-00378-5)
Supplement: Supplementary file 1 — Supplementary material [file 41391_2021_378_MOESM1_ESM.docx]

**SUPPLEMENTARY MATERIAL**

**The PRACTICAL Consortium:**

Rosalind Eeles ^1, 2^, Doug Easton ^3^, Zsofia Kote-Jarai ^1^, Ali Amin Al Olama ^3^, Sara Benlloch ^3^, Kenneth Muir ^4^, Graham Giles ^5, 6^, Fredrik Wiklund ^7^, Henrik Gronberg ^7^, Christopher Haiman ^8^, Johanna Schleutker ^9, 10^, Maren Weischer ^11^, Ruth C. Travis ^12^, David Neal ^13^, Paul Pharoah ^14^, Kay-Tee Khaw ^15^, Janet L. Stanford ^16, 17^, William J. Blot ^18^, Stephen Thibodeau ^19^, Christiane Maier ^20, 21^, Adam S. Kibel ^22, 23^, Cezary Cybulski ^24^, Lisa Cannon-Albright ^25^, Hermann Brenner ^26^, Jong Park ^27^, Radka Kaneva ^28^, Jyotnsa Batra ^29^, Manuel R. Teixeira ^30^, Hardev Pandha^31^

^1^ The Institute of Cancer Research, 15 Cotswold Road, Sutton, Surrey, SM2 5NG, UK, ^2^ Royal Marsden NHS Foundation Trust, Fulham and Sutton, London and Surrey, UK, ^3^ Centre for Cancer Genetic Epidemiology, Department of Public Health and Primary Care, University of Cambridge, Strangeways Laboratory, Worts Causeway, Cambridge, UK, ^4^ University of Warwick, Coventry, UK, ^5^ Cancer Epidemiology Centre, The Cancer Council Victoria, 1 Rathdowne street, Carlton Victoria, Australia, ^6^ Centre for Molecular, Environmental, Genetic and Analytic Epidemiology, The University of Melbourne, Victoria, Australia, ^7^ Department of Medical Epidemiology and Biostatistics, Karolinska Institute, Stockholm, Sweden, ^8^ Department of Preventive Medicine, Keck School of Medicine, University of Southern California/Norris Comprehensive Cancer Center, Los Angeles, California, USA, ^9^ Department of Medical Biochemistry and Genetics, University of Turku, Turku, Finland, ^10^ Institute of Biomedical Technology/BioMediTech, University of Tampere and FimLab Laboratories, Tampere, Finland, ^11^ Department of Clinical Biochemistry, Herlev Hospital, Copenhagen University Hospital, Herlev Ringvej 75, DK-2730 Herlev, Denmark, ^12^ Cancer Epidemiology Unit, Nuffield Department of Clinical Medicine, University of Oxford, Oxford, UK, ^13^ Surgical Oncology (Uro-Oncology: S4), University of Cambridge, Box 279, Addenbrooke’s Hospital, Hills Road, Cambridge, UK and Cancer Research UK Cambridge Research Institute, Li Ka Shing Centre, Cambridge, UK, ^14^ Centre for Cancer Genetic Epidemiology, Department of Oncology, University of Cambridge, Strangeways Laboratory, Worts Causeway, Cambridge, UK, ^15^ Cambridge Institute of Public Health, University of Cambridge, Forvie Site, Robinson Way, Cambridge CB2 0SR, ^16^ Division of Public Health Sciences, Fred Hutchinson Cancer Research Center, Seattle, Washington, USA, ^17^ Department of Epidemiology, School of Public Health, University of Washington, Seattle, Washington, USA, ^18^ International Epidemiology Institute, 1455 Research Blvd., Suite 550, Rockville, MD 20850, ^19^ Mayo Clinic, Rochester, Minnesota, USA, ^20^ Department of Urology, University Hospital Ulm, Germany, ^21^ Institute of Human Genetics University Hospital Ulm, Germany, ^22^ Brigham and Women's Hospital/Dana-Farber Cancer Institute, 45 Francis Street- ASB II-3, Boston, MA 02115, ^23^ Washington University, St Louis, Missouri, ^24^ International Hereditary Cancer Center, Department of Genetics and Pathology, Pomeranian Medical University, Szczecin, Poland, ^25^ Division of Genetic Epidemiology, Department of Medicine, University of Utah School of Medicine, ^26^ Division of Clinical Epidemiology and Aging Research, German Cancer Research Center, Heidelberg Germany, ^27^ Division of Cancer Prevention and Control, H. Lee Moffitt Cancer Center, 12902 Magnolia Dr., Tampa, Florida, USA, ^28^ Molecular Medicine Center and Department of Medical Chemistry and Biochemistry, Medical University - Sofia, 2 Zdrave St, 1431, Sofia, Bulgaria, ^29^ Australian Prostate Cancer Research Centre-Qld, Institute of Health and Biomedical Innovation and Schools of Life Science and Public Health, Queensland University of Technology, Brisbane, Australia, ^30^ Department of Genetics, Portuguese Oncology Institute, Porto, Portugal and Biomedical Sciences Institute (ICBAS), Porto University, Porto, Portugal, ^31^The University of Surrey, Guildford, Surrey, GU2 7XH.

**Funding for the CRUK study and PRACTICAL consortium:**

This work was supported by the Canadian Institutes of Health Research, European Commission's Seventh Framework Programme grant agreement n° 223175 (HEALTH-F2-2009-223175), Cancer Research UK Grants C5047/A7357, C1287/A10118, C5047/A3354, C5047/A10692, C16913/A6135, and The National Institute of Health (NIH) Cancer Post-Cancer GWAS initiative grant: No. 1 U19 CA 148537-01 (the GAME-ON initiative).

**COGS acknowledgement:**

This study would not have been possible without the contributions of the following: Per Hall (COGS); Douglas F. Easton, Paul Pharoah, Kyriaki Michailidou, Manjeet K. Bolla, Qin Wang (BCAC), Andrew Berchuck (OCAC), Rosalind A. Eeles, Douglas F. Easton, Ali Amin Al Olama, Zsofia Kote-Jarai, Sara Benlloch (PRACTICAL), Georgia Chenevix-Trench, Antonis Antoniou, Lesley McGuffog, Fergus Couch and Ken Offit (CIMBA), Joe Dennis, Alison M. Dunning, Andrew Lee, and Ed Dicks, Craig Luccarini and the staff of the Centre for Genetic Epidemiology Laboratory, Javier Benitez, Anna Gonzalez-Neira and the staff of the CNIO genotyping unit, Jacques Simard and Daniel C. Tessier, Francois Bacot, Daniel Vincent, Sylvie LaBoissière and Frederic Robidoux and the staff of the McGill University and Génome Québec Innovation Centre, Stig E. Bojesen, Sune F. Nielsen, Borge G. Nordestgaard, and the staff of the Copenhagen DNA laboratory, and Julie M. Cunningham, Sharon A. Windebank, Christopher A. Hilker, Jeffrey Meyer and the staff of Mayo Clinic Genotyping Core Facility

Funding for the iCOGS infrastructure came from: the European Community's Seventh Framework Programme under grant agreement n° 223175 (HEALTH-F2-2009-223175) (COGS), Cancer Research UK (C1287/A10118, C1287/A 10710, C12292/A11174, C1281/A12014, C5047/A8384, C5047/A15007, C5047/A10692), the National Institutes of Health (CA128978) and Post-Cancer GWAS initiative (1U19 CA148537, 1U19 CA148065 and 1U19 CA148112 - the GAME-ON initiative), the Department of Defence (W81XWH-10-1-0341), the Canadian Institutes of Health Research (CIHR) for the CIHR Team in Familial Risks of Breast Cancer, Komen Foundation for the Cure, the Breast Cancer Research Foundation, and the Ovarian Cancer Research Fund.

**Supplementary Figure 1**

Manhattan plot of associations for 160 identified prostate cancer susceptibility loci. Genome-wide significance level is represented by the red line *P*<5 x10^−8^. The blue line is *P* = 1x10^−5^.


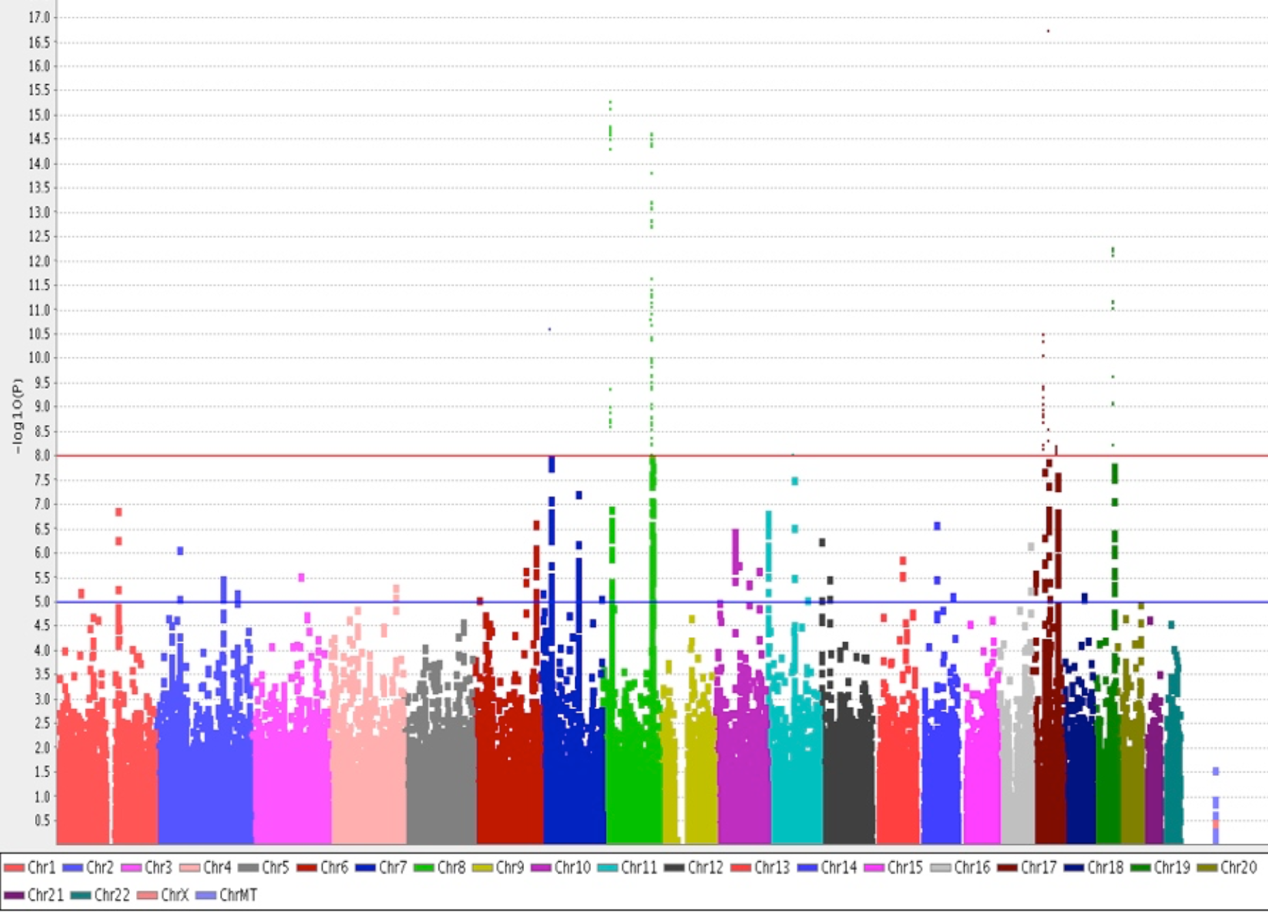


**Supplementary table 1. Summary results for 160 prostate cancer susceptibility loci before LD pruning**

| **Marker** | **Locus** | **Position** | **Alleles^a^** | **EAF^b^** | **OR^c^ (95% CI)** | **P-value** | **P_adj_value^d^** | **Annotation, nearby genes** |  |
| --- | --- | --- | --- | --- | --- | --- | --- | --- | --- |
| rs16902147 | 8q24.21 | 128474254 | GA | 0.07 | 1.86 (1.56-2.23) | 1.214E-11 | 3.525E-8 | CASC8 |  |
| rs17467139 | 8q24.21 | 128481192 | GA | 0.07 | 1.84 (1.53-2.21) | 1.033E-10 | 2.473E-7 | CASC8 |  |
| rs78654475 | 8q24.21 | 128477827 | AC | 0.07 | 1.82 (1.52-2.19) | 1.217E-10 | 2.875E-7 | CASC8 |  |
| rs79012498 | 8q24.21 | 128222502 | GA | 0.08 | 1.81 (1.53-2.15) | 1.507E-11 | 4.26E-8 | Intergenic |  |
| rs16902149 | 8q24.21 | 128476287 | CG | 0.07 | 1.80 (1.50-2.16) | 2.316E-10 | 5.185E-7 | CASC8 |  |
| rs75078154 | 8q24.21 | 128475507 | GA | 0.07 | 1.80 (1.50-2.16) | 2.316E-10 | 5.185E-7 | CASC8 |  |
| rs7822052 | 8q24.21 | 128492909 | TA | 0.07 | 1.79 (1.49-2.15) | 3.746E-10 | 7.874E-7 | CASC8, POU5F1B |  |
| rs11650494 | 17q21.32 | 44700185 | AG | 0.06 | 1.76 (1.46-2.12) | 2.817E-9 | 3.981E-6 | Intergenic |  |
| rs11650886 | 17q21.33 | 44804192 | AG | 0.06 | 1.74 (1.45-2.10) | 4.948E-9 | 6.717E-6 | RP11-81K2.1, RP11-1079K10.2, RP11-1079K10.3 | |
| rs7216993 | 17q21.33 | 44809262 | GA | 0.06 | 1.74 (1.45-2.10) | 4.948E-9 | 6.717E-6 | RP11-81K2.1, RP11-1079K10.2 | |
| rs76694703 | 8q24.21 | 128523466 | GA | 0.07 | 1.74 (1.45-2.08) | 2.08E-9 | 3.265E-6 | CASC8 |  |
| rs58809953 | 8q24.21 | 128477243 | AG | 0.07 | 1.71 (1.43-2.04) | 2.855E-9 | 4.001E-6 | CASC8 |  |
| rs75753382 | 8q24.21 | 128510730 | AG | 0.07 | 1.70 (1.42-2.04) | 5.916E-9 | 7.847E-6 | CASC8 |  |
| rs2074187 | 17q21.32 | 43285358 | AC | 0.07 | 1.66 (1.38-1.98) | 3.743E-8 | 3.752E-5 | SP6 |  |
| rs7832031 | 8q24.21 | 128586134 | AG | 0.22 | 1.49 (1.35-1.65) | 2.374E-15 | 3.003E-11 | Intergenic |  |
| rs2121630 | 8q24.21 | 128547342 | AC | 0.16 | 1.49 (1.33-1.67) | 2.047E-11 | 5.691E-8 | CASC8 |  |
| rs10808558 | 8q24.21 | 128570332 | AG | 0.23 | 1.48 (1.34-1.63) | 2.604E-15 | 3.003E-11 | Intergenic |  |
| rs4314621 | 8q24.21 | 128587197 | GA | 0.23 | 1.48 (1.34-1.63) | 4.385E-15 | 3.98E-11 | Intergenic |  |
| rs7812894 | 8q24.21 | 128589661 | TA | 0.23 | 1.48 (1.34-1.63) | 3.857E-15 | 3.694E-11 | Intergenic |  |
| rs13255059 | 8q24.21 | 128599798 | AG | 0.22 | 1.47 (1.34-1.63) | 1.507E-14 | 1.237E-10 | Intergenic |  |
| rs9297759 | 8q24.21 | 128588352 | AC | 0.22 | 1.46 (1.32-1.61) | 6.386E-14 | 4.787E-10 | Intergenic |  |
| rs11995378 | 8q24.21 | 128565593 | AG | 0.22 | 1.45 (1.32-1.60) | 8.096E-14 | 5.568E-10 | CASC8 |  |
| rs7824868 | 8q24.21 | 128593596 | AG | 0.22 | 1.45 (1.32-1.60) | 8.514E-14 | 5.568E-10 | Intergenic |  |
| rs4242382 | 8q24.21 | 128586755 | AG | 0.22 | 1.45 (1.31-1.60) | 1.882E-13 | 1.081E-9 | Intergenic |  |
| rs4515512 | 8q24.21 | 128601580 | AG | 0.22 | 1.45 (1.31-1.60) | 1.943E-13 | 1.081E-9 | Intergenic |  |
| rs7812429 | 8q24.21 | 128589355 | AG | 0.22 | 1.45 (1.31-1.60) | 1.447E-13 | 8.909E-10 | Enhancer |  |
| rs7814837 | 8q24.21 | 128591384 | AC | 0.22 | 1.45 (1.31-1.60) | 1.547E-13 | 9.198E-10 | Intergenic |  |
| rs12275055 | 11q13.3 | 68737934 | GA | 0.13 | 1.44 (1.27-1.63) | 9.286E-9 | 1.119E-5 | Intergenic |  |
| rs9656816 | 8q24.21 | 128603836 | GA | 0.17 | 1.43 (1.28-1.60) | 2.247E-10 | 5.165E-7 | Intergenic |  |
| rs10109700 | 8q24.21 | 128555146 | AG | 0.23 | 1.42 (1.29-1.56) | 2.35E-12 | 9.646E-9 | CASC8 |  |
| rs4871801 | 8q24.21 | 128553095 | AG | 0.23 | 1.42 (1.29-1.56) | 2.35E-12 | 9.646E-9 | CASC8 |  |
| rs4871802 | 8q24.21 | 128554821 | CA | 0.23 | 1.42 (1.29-1.56) | 2.35E-12 | 9.646E-9 | CASC8 |  |
| rs6470519 | 8q24.21 | 128553405 | AC | 0.23 | 1.42 (1.29-1.56) | 2.35E-12 | 9.646E-9 | CASC8 |  |
| rs7818556 | 8q24.21 | 128553581 | GA | 0.23 | 1.42 (1.29-1.56) | 2.35E-12 | 9.646E-9 | CASC8 |  |
| rs12795301 | 11q13.3 | 68748861 | AC | 0.13 | 1.42 (1.25-1.61) | 2.786E-8 | 2.894E-5 | Intergenic |  |
| rs1447295 | 8q24.21 | 128554220 | AC | 0.23 | 1.41 (1.28-1.55) | 5.26E-12 | 2.015E-8 | CASC8 |  |
| rs4871813 | 8q24.21 | 128617123 | AC | 0.22 | 1.41 (1.28-1.55) | 7.169E-12 | 2.332E-8 | Intergenic |  |
| rs6470529 | 8q24.21 | 128613993 | CG | 0.22 | 1.41 (1.28-1.55) | 6.904E-12 | 2.332E-8 | Intergenic |  |
| rs9643226 | 8q24.21 | 128563663 | GC | 0.23 | 1.41 (1.28-1.55) | 4.825E-12 | 1.891E-8 | CASC8 |  |
| rs1447296 | 8q24.21 | 128564541 | AG | 0.23 | 1.40 (1.27-1.55) | 8.454E-12 | 2.603E-8 | CASC8 |  |
| rs1160267 | 8p21.2 | 23585466 | GA | 0.53 | 1.38 (1.28-1.50) | 5.183E-16 | 3.003E-11 | Intergenic |  |
| rs1512268 | 8p21.2 | 23582408 | AG | 0.53 | 1.38 (1.28-1.50) | 5.183E-16 | 3.003E-11 | Intergenic |  |
| rs995432 | 8p21.2 | 23578796 | GA | 0.53 | 1.38 (1.28-1.50) | 7.575E-16 | 3.003E-11 | Intergenic |  |
| rs13256300 | 8p21.2 | 23558533 | AG | 0.51 | 1.37 (1.27-1.48) | 2.613E-15 | 3.003E-11 | Intergenic |  |
| rs13256366 | 8p21.2 | 23558604 | AG | 0.51 | 1.37 (1.27-1.48) | 2.235E-15 | 3.003E-11 | Intergenic |  |
| rs1398238 | 8p21.2 | 23572976 | CG | 0.51 | 1.37 (1.27-1.48) | 2.235E-15 | 3.003E-11 | Intergenic |  |
| rs1398239 | 8p21.2 | 23557674 | CA | 0.51 | 1.37 (1.27-1.48) | 2.613E-15 | 3.003E-11 | Intergenic |  |
| rs1398240 | 8p21.2 | 23557551 | CA | 0.51 | 1.37 (1.27-1.48) | 2.613E-15 | 3.003E-11 | Intergenic |  |
| rs1512271 | 8p21.2 | 23572445 | AT | 0.51 | 1.37 (1.27-1.48) | 2.613E-15 | 3.003E-11 | Intergenic |  |
| rs2315144 | 8p21.2 | 23571472 | CG | 0.51 | 1.37 (1.27-1.48) | 1.717E-15 | 3.003E-11 | Intergenic |  |
| rs4872171 | 8p21.2 | 23570234 | AG | 0.51 | 1.37 (1.27-1.48) | 2.235E-15 | 3.003E-11 | Intergenic |  |
| rs4872172 | 8p21.2 | 23570288 | AC | 0.51 | 1.37 (1.27-1.48) | 2.235E-15 | 3.003E-11 | Intergenic |  |
| rs4872175 | 8p21.2 | 23590747 | AG | 0.53 | 1.37 (1.27-1.48) | 1.989E-15 | 3.003E-11 | NKX3.1 |  |
| rs7830220 | 8p21.2 | 23555627 | GA | 0.51 | 1.37 (1.27-1.48) | 3.128E-15 | 3.172E-11 | Intergenic |  |
| rs13265330 | 8p21.2 | 23581488 | GA | 0.53 | 1.37 (1.26-1.48) | 5.092E-15 | 4.39E-11 | Intergenic |  |
| rs4242386 | 8q24.21 | 128610274 | GA | 0.23 | 1.37 (1.25-1.51) | 1.505E-10 | 3.507E-7 | Intergenic |  |
| rs8180905 | 8q24.21 | 128608005 | AG | 0.27 | 1.36 (1.24-1.49) | 4.218E-11 | 1.069E-7 | Intergenic |  |
| rs4871798 | 8q24.21 | 128549145 | AG | 0.28 | 1.33 (1.22-1.46) | 3.105E-10 | 6.716E-7 | CASC8 |  |
| rs7013278 | 8q24.21 | 128484074 | AG | 0.38 | 1.32 (1.21-1.43) | 3.648E-11 | 9.387E-8 | CASC8, POU5F1B |  |
| rs2166689 | 8q24.21 | 128547496 | CA | 0.34 | 1.31 (1.21-1.43) | 3.764E-10 | 7.874E-7 | CASC8 |  |
| rs7014346 | 8q24.21 | 128493974 | AG | 0.38 | 1.31 (1.21-1.42) | 9.771E-11 | 2.373E-7 | CASC8, POU5F1B |  |
| rs921146 | 8q24.21 | 128544367 | CA | 0.34 | 1.31 (1.20-1.43) | 4.321E-10 | 8.562E-7 | CASC8 |  |
| rs10505476 | 8q24.21 | 128477298 | AG | 0.36 | 1.31 (1.20-1.42) | 3.116E-10 | 6.716E-7 | CASC8 |  |
| rs10956373 | 8q24.21 | 128559758 | GA | 0.33 | 1.29 (1.19-1.41) | 4.303E-9 | 5.935E-6 | CASC8 |  |
| rs4872174 | 8p21.2 | 23581156 | GA | 0.47 | 1.28 (1.19-1.39) | 4.199E-10 | 8.547E-7 | Intergenic |  |
| rs10505474 | 8q24.21 | 128486686 | AG | 0.47 | 1.28 (1.18-1.39) | 8.737E-10 | 1.637E-6 | CASC8 |  |
| rs10808556 | 8q24.21 | 128482329 | GA | 0.47 | 1.28 (1.18-1.38) | 1.091E-9 | 1.96E-6 | CASC8 |  |
| rs10956370 | 8q24.21 | 128493909 | CA | 0.47 | 1.28 (1.18-1.38) | 1.615E-9 | 2.757E-6 | CASC8, POU5F1B |  |
| rs3847137 | 8q24.21 | 128483680 | GA | 0.47 | 1.28 (1.18-1.38) | 1.03E-9 | 1.869E-6 | CASC8 |  |
| rs4871896 | 8p21.2 | 23546117 | GA | 0.45 | 1.28 (1.18-1.38) | 1.285E-9 | 2.261E-6 | FAM60DP |  |
| rs4872170 | 8p21.2 | 23556798 | CG | 0.45 | 1.28 (1.18-1.38) | 1.01E-9 | 1.852E-6 | Intergenic |  |
| rs10956367 | 8q24.21 | 128492725 | AG | 0.47 | 1.27 (1.18-1.38) | 2.286E-9 | 3.284E-6 | CASC8, POU5F1B |  |
| rs10956366 | 8q24.21 | 128492672 | GA | 0.47 | 1.27 (1.18-1.38) | 2.254E-9 | 3.265E-6 | CASC8, POU5F1B |  |
| rs11773905 | 8p21.2 | 23567108 | GA | 0.45 | 1.27 (1.18-1.38) | 2.064E-9 | 3.265E-6 | Intergenic |  |
| rs11784983 | 8q24.21 | 128490529 | AG | 0.47 | 1.27 (1.18-1.38) | 1.713E-9 | 2.896E-6 | CASC8 |  |
| rs11998706 | 8q24.21 | 128491279 | GC | 0.47 | 1.27 (1.18-1.38) | 2.254E-9 | 3.265E-6 | CASC8, POU5F1B |  |
| rs12678562 | 8q24.21 | 128491669 | AG | 0.47 | 1.27 (1.18-1.38) | 2.254E-9 | 3.265E-6 | CASC8, POU5F1B |  |
| rs4242402 | 8p21.2 | 23569675 | AG | 0.45 | 1.27 (1.18-1.38) | 1.821E-9 | 3.048E-6 | Intergenic |  |
| rs4276648 | 8q24.21 | 128496554 | AG | 0.47 | 1.27 (1.18-1.38) | 2.109E-9 | 3.265E-6 | CASC8, POU5F1B |  |
| rs4871022 | 8q24.21 | 128496902 | GA | 0.47 | 1.27 (1.18-1.38) | 2.254E-9 | 3.265E-6 | CASC8, POU5F1B |  |
| rs7013328 | 8q24.21 | 128493092 | GA | 0.47 | 1.27 (1.18-1.38) | 2.254E-9 | 3.265E-6 | CASC8, POU5F1B |  |
| rs7018367 | 8q24.21 | 128494064 | GA | 0.47 | 1.27 (1.18-1.38) | 2.254E-9 | 3.265E-6 | CASC8, POU5F1B |  |
| rs7018368 | 8q24.21 | 128494114 | AG | 0.47 | 1.27 (1.18-1.38) | 2.254E-9 | 3.265E-6 | CASC8, POU5F1B |  |
| rs7837328 | 8q24.21 | 128492309 | AG | 0.47 | 1.27 (1.18-1.38) | 2.254E-9 | 3.265E-6 | CASC8, POU5F1B |  |
| rs7837626 | 8q24.21 | 128492523 | AG | 0.47 | 1.27 (1.18-1.38) | 2.254E-9 | 3.265E-6 | CASC8, POU5F1B |  |
| rs7837644 | 8q24.21 | 128492580 | TA | 0.47 | 1.27 (1.18-1.38) | 2.254E-9 | 3.265E-6 | CASC8, POU5F1B |  |
| rs7837706 | 8q24.21 | 128492365 | GC | 0.47 | 1.27 (1.18-1.38) | 2.254E-9 | 3.265E-6 | CASC8, POU5F1B |  |
| rs871135 | 8q24.21 | 128495575 | CA | 0.47 | 1.27 (1.18-1.38) | 2.254E-9 | 3.265E-6 | CASC8, POU5F1B |  |
| rs4543510 | 8q24.21 | 128614800 | AG | 0.33 | 1.27 (1.17-1.38) | 2.85E-8 | 2.908E-5 | Intergenic |  |
| rs6985504 | 8q24.21 | 128565958 | AG | 0.35 | 1.27 (1.17-1.38) | 1.482E-8 | 1.649E-5 | CASC8 |  |
| rs10808557 | 8q24.21 | 128496487 | GA | 0.47 | 1.27 (1.17-1.37) | 2.894E-9 | 4.024E-6 | CASC8, POU5F1B |  |
| rs11135762 | 8p21.2 | 23547136 | GA | 0.45 | 1.27 (1.17-1.37) | 2.463E-9 | 3.509E-6 | RP11-583M2.2 |  |
| rs4793976 | 17q21.32 | 44135496 | GA | 0.39 | 1.26 (1.17-1.37) | 1.216E-8 | 1.397E-5 | LINC02086 |  |
| rs10956368 | 8q24.21 | 128492832 | AG | 0.46 | 1.26 (1.16-1.36) | 9.483E-9 | 1.128E-5 | CASC8, POU5F1B |  |
| rs1447293 | 8q24.21 | 128541502 | GA | 0.50 | 1.26 (1.16-1.36) | 5.639E-9 | 7.596E-6 | CASC8 |  |
| rs3760511 | 17q12 | 33180426 | CA | 0.49 | 1.26 (1.16-1.36) | 7.107E-9 | 9.01E-6 | HNF1B |  |
| rs10956372 | 8q24.21 | 128539438 | TA | 0.44 | 1.25 (1.16-1.35) | 2.82E-8 | 2.894E-5 | CASC8 |  |
| rs67143603 | 17q12 | 33182344 | GA | 0.49 | 1.25 (1.16-1.35) | 1.885E-8 | 2.044E-5 | HNF1B |  |
| rs6501437 | 17q24.3 | 66631567 | CG | 0.51 | 1.24 (1.15-1.34) | 3.225E-8 | 3.27E-5 | CASC17 |  |
| rs6501438 | 17q24.3 | 66631755 | AG | 0.51 | 1.24 (1.15-1.34) | 3.912E-8 | 3.899E-5 | CASC17 |  |
| rs1567669 | 8p21.2 | 23594478 | AG | 0.47 | 1.23 (1.19-1.39) | 4.29E-10 | 8.562E-7 | NKX3.1 |  |
| rs4793529 | 17q24.3 | 66630231 | GA | 0.47 | 0.80 (0.74-0.87) | 2.416E-8 | 2.557E-5 | CASC17 |  |
| rs4871790 | 8q24.21 | 128510716 | CA | 0.45 | 0.80 (0.74-0.87) | 1.658E-8 | 1.809E-5 | CASC8 |  |
| rs587948 | 8q24.21 | 128410862 | CA | 0.34 | 0.80 (0.74-0.87) | 2.819E-8 | 2.894E-5 | CASC8 |  |
| rs1668875 | 8q24.21 | 128410285 | CG | 0.33 | 0.80 (0.74-0.86) | 2.417E-8 | 2.557E-5 | CASC8 |  |
| rs17765332 | 17q24.3 | 66618336 | GA | 0.47 | 0.80 (0.74-0.86) | 6.648E-9 | 8.553E-6 | CASC17 |  |
| rs17765344 | 17q24.3 | 66618469 | GA | 0.47 | 0.80 (0.74-0.86) | 8.209E-9 | 9.967E-6 | CASC17 |  |
| rs1859962 | 17q24.3 | 66620348 | AC | 0.47 | 0.80 (0.74-0.86) | 9.644E-9 | 1.139E-5 | CASC17 |  |
| rs623401 | 8q24.21 | 128410909 | CG | 0.33 | 0.80 (0.74-0.86) | 2.684E-8 | 2.822E-5 | CASC8 |  |
| rs7222314 | 17q24.3 | 66616533 | GA | 0.47 | 0.80 (0.74-0.86) | 6.845E-9 | 8.742E-6 | CASC17 |  |
| rs8068266 | 17q24.3 | 66628530 | GA | 0.47 | 0.80 (0.74-0.86) | 6.455E-9 | 8.368E-6 | CASC17 |  |
| rs8071558 | 17q24.3 | 66619268 | GC | 0.47 | 0.80 (0.74-0.86) | 9.349E-9 | 1.119E-5 | CASC17 |  |
| rs8072254 | 17q24.3 | 66619411 | GA | 0.47 | 0.80 (0.74-0.86) | 7.299E-9 | 9.12E-6 | CASC17 |  |
| rs8072735 | 17q24.3 | 66628808 | GA | 0.47 | 0.80 (0.74-0.86) | 6.455E-9 | 8.368E-6 | CASC17 |  |
| rs984434 | 17q24.3 | 66619722 | GA | 0.47 | 0.80 (0.74-0.86) | 7.299E-9 | 9.12E-6 | CASC17 |  |
| rs9889335 | 17q24.3 | 66626741 | CA | 0.47 | 0.80 (0.74-0.86) | 7.974E-9 | 9.82E-6 | CASC17 |  |
| rs9911515 | 17q24.3 | 66626952 | GA | 0.47 | 0.80 (0.74-0.86) | 7.974E-9 | 9.82E-6 | CASC17 |  |
| rs991429 | 17q24.3 | 66621368 | AG | 0.47 | 0.80 (0.74-0.86) | 8.209E-9 | 9.967E-6 | CASC17 |  |
| rs12601991 | 17q12 | 33175745 | CA | 0.42 | 0.79 (0.73-0.86) | 6.068E-9 | 7.986E-6 | HNF1B |  |
| rs2739459 | 19q13.33 | 56060886 | GA | 0.42 | 0.79 (0.73-0.86) | 2.186E-8 | 2.355E-5 | KLK2 |  |
| rs11263762 | 17q12 | 33176038 | GA | 0.40 | 0.79 (0.73-0.85) | 1.103E-9 | 1.961E-6 | HNF1B |  |
| rs11651755 | 17q12 | 33173952 | GA | 0.35 | 0.79 (0.73-0.85) | 1.998E-9 | 3.265E-6 | HNF1B |  |
| rs7405696 | 17q12 | 33176148 | GC | 0.40 | 0.79 (0.73-0.85) | 1.599E-9 | 2.757E-6 | HNF1B |  |
| rs10956359 | 8q24.21 | 128411336 | GA | 0.25 | 0.78 (0.72-0.86) | 3.72E-8 | 3.751E-5 | CASC8 |  |
| rs757138 | 7p15.2 | 27955927 | CA | 0.26 | 0.78 (0.72-0.85) | 1.114E-8 | 1.289E-5 | JAZF1 |  |
| rs4239217 | 17q12 | 33173100 | GA | 0.27 | 0.77 (0.71-0.84) | 1.347E-9 | 2.345E-6 | HNF1B |  |
| rs7501939 | 17q12 | 33175269 | AG | 0.27 | 0.77 (0.71-0.83) | 6.317E-10 | 1.224E-6 | HNF1B |  |
| rs8064454 | 17q12 | 33175698 | AC | 0.32 | 0.77 (0.71-0.83) | 8.477E-11 | 2.088E-7 | HNF1B |  |
| rs17464492 | 8q24.21 | 128412048 | GA | 0.21 | 0.77 (0.70-0.85) | 2.275E-8 | 2.436E-5 | CASC8 |  |
| rs10107982 | 8q24.21 | 128387937 | GA | 0.21 | 0.77 (0.70-0.84) | 1.052E-8 | 1.227E-5 | CASC8 |  |
| rs10807843 | 7p15.2 | 27958915 | GA | 0.20 | 0.77 (0.70-0.84) | 1.34E-8 | 1.5E-5 | JAZF1 |  |
| rs11971132 | 7p15.2 | 27952391 | GC | 0.20 | 0.77 (0.70-0.84) | 1.34E-8 | 1.5E-5 | JAZF1 |  |
| rs11982766 | 7p15.2 | 27950306 | AG | 0.20 | 0.77 (0.70-0.84) | 1.34E-8 | 1.5E-5 | JAZF1 |  |
| rs67746093 | 7p15.2 | 27952049 | CG | 0.20 | 0.77 (0.70-0.84) | 1.34E-8 | 1.5E-5 | JAZF1 |  |
| rs757136 | 7p15.2 | 27955464 | GA | 0.20 | 0.77 (0.70-0.84) | 1.549E-8 | 1.701E-5 | JAZF1 |  |
| rs11657964 | 17q12 | 33174879 | AG | 0.27 | 0.77 (0.70-0.83) | 4.214E-10 | 8.547E-7 | HNF1B |  |
| rs7405776 | 17q12 | 33167134 | AG | 0.26 | 0.77 (0.70-0.83) | 8.439E-10 | 1.599E-6 | HNF1B |  |
| rs11651052 | 17q12 | 33176493 | AG | 0.32 | 0.76 (0.70-0.83) | 4.4E-11 | 1.099E-7 | HNF1B |  |
| rs757210 | 17q12 | 33170628 | AG | 0.25 | 0.76 (0.70-0.83) | 3.791E-10 | 7.874E-7 | HNF1B |  |
| rs2005705 | 17q12 | 33170413 | AG | 0.31 | 0.76 (0.70-0.82) | 3.255E-11 | 8.503E-8 | HNF1B |  |
| rs2040668 | 7p15.2 | 27925298 | CA | 0.19 | 0.76 (0.69-0.84) | 1.053E-8 | 1.227E-5 | JAZF1 |  |
| rs266876 | 19q13.33 | 56052629 | GA | 0.19 | 0.76 (0.69-0.84) | 2.745E-8 | 2.868E-5 | KLK3 |  |
| rs10505477 | 8q24.21 | 128476625 | GA | 0.46 | 0.74 (0.69-0.80) | 6.105E-14 | 4.785E-10 | CASC8 |  |
| rs12682374 | 8q24.21 | 128480129 | CG | 0.45 | 0.74 (0.69-0.80) | 8.72E-14 | 5.568E-10 | CASC8 |  |
| rs10486567 | 7p15.2 | 27943088 | AG | 0.23 | 0.74 (0.68-0.81) | 2.424E-11 | 6.43E-8 | JAZF1 |  |
| rs67152137 | 7p15.2 | 27942444 | GC | 0.23 | 0.74 (0.68-0.81) | 2.424E-11 | 6.43E-8 | JAZF1 |  |
| rs7808935 | 7p15.2 | 27943888 | GA | 0.23 | 0.74 (0.68- 0.81) | 2.424E-11 | 6.43E-8 | JAZF1 |  |
| rs6983267 | 8q24.21 | 128482487 | AC | 0.43 | 0.73 (0.68-0.79) | 3.114E-15 | 3.172E-11 | CASC8 |  |
| rs2659122 | 19q13.33 | 56054838 | GA | 0.17 | 0.73 (0.66-0.81) | 8.976E-10 | 1.664E-6 | KLK3 |  |
| rs266849 | 19p13.33 | 56040902 | GA | 0.12 | 0.72 (0.65-0.81) | 1.531E-8 | 1.692E-5 | AC011523.2 |  |
| rs2659051 | 19p13.33 | 56037380 | GC | 0.12 | 0.72 (0.64-0.80) | 5.894E-9 | 7.847E-6 | AC011523.2 |  |
| rs62113216 | 19q13.33 | 56056615 | AT | 0.10 | 0.67 (0.60-0.76) | 2.387E-10 | 5.277E-7 | KLK3 region |  |
| rs1058205 | 19q13.33 | 56055210 | GA | 0.12 | 0.67 (0.60-0.75) | 7.493E-13 | 3.691E-9 | KLK3 |  |
| rs2569735 | 19q13.33 | 56056081 | AG | 0.11 | 0.67 (0.60-0.75) | 8.976E-12 | 2.668E-8 | KLK3 region |  |
| rs2735839 | 19q13.33 | 56056435 | AG | 0.11 | 0.67 (0.60-0.75) | 8.976E-12 | 2.668E-8 | KLK3 region |  |
| rs174776 | 19q13.33 | 56051664 | AG | 0.07 | 0.63 (0.55-0.71) | 6.666E-12 | 2.307E-8 | KLK3 |  |
| rs266878 | 19q13.33 | 56050926 | CG | 0.07 | 0.62 (0.54-0.71) | 7.052E-12 | 2.332E-8 | KLK3 |  |
| rs78177998 | 19q13.33 | 56037076 | GA | 0.06 | 0.62 (0.53-0.72) | 8.045E-10 | 1.541E-6 | KLK13e |  |
| rs17632542 | 19q13.33 | 56053569 | GA | 0.06 | 0.59 (0.51-0.68) | 5.945E-13 | 3.015E-9 | KLK3 |  |
| rs62113212 | 19q13.33 | 56052652 | AG | 0.06 | 0.59 (0.51-0.68) | 5.447E-13 | 2.846E-9 | KLK3 |  |

^a^Effect allele/Other allele

^b^Effect allele frequency in cases

^c^Per-allele odds ratio for the effect allele

^d^Adjusted for false discovery rate (FDR) using Benjamini-Hochberg method

Supplementary table 2. Association of the identified 10 novel variants with malignant neoplasm of prostate in FinnGen and UKBB (UK Biobank) population-based data.

| rs ID | **FinnGen p-value** | **FinnGen N case/control** | **UKBB p-value** | **UKBB N case/control** |
| --- | --- | --- | --- | --- |
| rs16902147 | 3.6e-12 | 3282 / 55968 | 8.8e-5 | 6321 / 354873 |
| **rs79012498** | 7.0e-15 | 3282 / 55968 | 3.1e-3 | 6321 / 354873 |
| rs58809953 | 3.7e-8 | 1824 / 40413 | 2.5e-7 | 6321 / 354873 |
| **rs2074187** | 2.8e-8 | 1824 / 40413 | 8.4e-1 | 6321 / 354873 |
| rs12795301 | 1.9e-10 | 3282 / 55968 | 5.0e-28 | 6321 / 354873 |
| rs995432 | 1.4e-6 | 3282 / 55968 | 1.3e-16 | 6321 / 354873 |
| rs4871790 | 1.4e-8 | 3282 / 55968 | 1.7e-19 | 6321 / 354873 |
| rs587948 | 8.5e-8 | 3282 / 55968 | 3.4e-11 | 6321 / 354873 |
| rs2739459 | 6.0e-2 | 3282 / 55968 | 2.3e-7 | 6321 / 354873 |
| rs757138 | 5.3e-6 | 3282 / 55968 | 6.0e-8 | 6321 / 354873 |

N, number of samples

Supplementary table 3. Summary of published susceptibility variants of aggressive prostate cancer

| **Gene** | **Variant** | **OR/HR** | **95% CI** | **p value** | **Aggressive PC defined as** | **Reference** |
| --- | --- | --- | --- | --- | --- | --- |
| HOXB13-CIP2A | rs138213197 -rs2278911 | 2.31 | 1.1-4.8 | 0.025 | Gleason score ≥ 8 | Sipeky 2018 |
| HERC2 | rs6497287 | 1.46 | 1.10–1.94 | 0.004 | more aggressive PC: diagnostic PSA ≥20ng/mL or 50ng/mL, regional/distant stage, Gleason score ≥7 (3+4), recurrence/progression event and/or prostate cancer-specific death; | FitzGerald 2011 |
| ANO7 | rs77559646 | 1.40 | 1.09–1.78 | 0.04 | Gleason grade ≥ 8 | Kaikkonen 2018 |
| DAB2IP | rs1571801 | 1.36 | 1.13-1.63 | 0.001 | pathologic stage T3/T4, N+, M+, Gleason score of 7 or more, or preoperative serum PSA of at least 20 ng/mL. | Duggan 2007 |
| TNFSF10 | rs3774315 | 1.25 | 1.08–1.46 | 0.002 | less aggressive PC | FitzGerald 2011 |
| PCAT19 | rs11672691 | 1.12 | 1.03-1.21 | 1.4x10^-8^ | Gleason score ≥ 8 | AL Olama 2013 |
| PDLIM5 | rs17021918 | 0.93 | 0.89–1.16 | 0.789 | less aggressive PC | Jinga 2016 |
| TERT | rs2736100 | 0.81 | 0.66-0.99 | 0.037 | decreased risk of aggressive PC (aggresive PC defined as diagnosed with N1 or M1) | Wu D 2015 |
| KLK3 | rs2735839 | 0.77 | 0.69-0.87 | 1.042x10^-5^ | high grade PC | Helfand 2015 |
| TERT | rs10069690 | 0.76 | 0.59-0.97 | 0.030 | decreased risk of aggressive PC (aggresive PC defined as diagnosed with N1 or M1) | Wu D 2015 |
| THADA | rs1465618 | 1.37 | 1.06–1.76 | 0.016 | less aggressive PC | Jinga 2016 |
| EHBP1 | rs721048 | 1.43 | 1.05–1.93 | 0.019 | less aggressive PC | Jinga 2016 |
| RASA1 | rs35148638 | - | - | 6.49×10^-9^ | high Gleason, aggressive PC | Berndt, 2015 |
| NAALADL | rs78943174 | - | - | 4.18×10^-8^ | high Gleason score | Berndt, 2015 |
| LSAMP | deletion | - | - | - | rapid disease progression | Petrovics 2015 |
| KLK3 | rs17632542 | - | - | 1.4×10^−5^ | PSA at diagnosis | Sullivan 2015 |
| ARVCF | rs5993891 | /0.52 | 0.29–0.93 | 0.03 | PC specific mortality | Penney KL, 2015 |
| *C2orf43* | rs13385191 | /1.36 | 1.02-1.81 | 0.03 | biochemical recurrence | Sullivan 2015 |
| *C2orf43* | rs13385191 | /1.28 | 1.02-1.60 | 0.02 | castrate metastasis | Sullivan 2015 |
| *RFX6/GPRC6A* | rs339331 | /1.45 | 1.03-2.02 | 0.02 | biochemical recurrence | Sullivan 2015 |
| *AFM,RASSF6* | rs1894292 | /1.25 | 1.01-1.54 | 0.03 | castrate metastasis | Sullivan 2015 |
| MSMB | rs17178655 | /0.73 | 0.55-0.97 | 0.03 | castrate metastasis | Sullivan 2015 |
| *OSTF1P1* | rs11067228 | /0.74 | 0.60-0.93 | 0.009 | castrate metastasis | Sullivan 2015 |
| *TAF1B:GRHL1* | rs11902236 | /0.78 | 0.62-0.98 | 0.03 | prostate cancer specific mortality | Sullivan 2015 |
| *EEFSEC* | rs4857841 | /0.78 | 0.62-0.98 | 0.04 | prostate cancer specific mortality | Sullivan 2015 |

References:

[Synergistic interaction of *HOXB13* and *CIP2A* predispose to aggressive prostate cancer](https://www.ncbi.nlm.nih.gov/pmc/articles/PMC6719560/)

Csilla **Sipeky**, Ping Gao, Qin Zhang, Liang Wang, Otto Ettala, Kirsi M Talala, Teuvo L.J. Tammela, Anssi Auvinen, Fredrik Wiklund, Gong-Hong Wei, Johanna Schleutker

Clin Cancer Res. Author manuscript; available in PMC 2019 Sep 3.

Published in final edited form as: Clin Cancer Res. 2018 Dec 15; 24(24): 6265–6276. Published online 2018 Sep 4. doi: 10.1158/1078-0432.CCR-18-0444

PMCID: PMC6719560

[Genome-wide association study identifies a genetic variant associated with risk for more aggressive prostate cancer](https://www.ncbi.nlm.nih.gov/pmc/articles/PMC3111761/)

Liesel M. **FitzGerald**, Erika M. Kwon, Matthew P. Conomos, Suzanne Kolb, Sarah K. Holt, David Levine, Ziding Feng, Elaine A. Ostrander, Janet L. Stanford

Cancer Epidemiol Biomarkers Prev. Author manuscript; available in PMC 2012 Jun 1.

Published in final edited form as: Cancer Epidemiol Biomarkers Prev. 2011 Jun; 20(6): 1196–1203. Published online 2011 Apr 5. doi: 10.1158/1055-9965.EPI-10-1299 PMCID: PMC3111761

[ANO7 is associated with aggressive prostate cancer](https://www.ncbi.nlm.nih.gov/pmc/articles/PMC6589920/)

Elina **Kaikkonen**, Tommi Rantapero, Qin Zhang, Pekka Taimen, Virpi Laitinen, Markku Kallajoki, Dhanaprakash Jambulingam, Otto Ettala, Juha Knaapila, Peter J. Boström, Gudrun Wahlström, Csilla Sipeky, Juha‐Pekka Pursiheimo, Teuvo Tammela, Pirkko‐Liisa Kellokumpu‐Lehtinen, PRACTICAL Consortium, Vidal Fey, Lovise Maehle, Fredrik Wiklund, Gong‐Hong Wei, Johanna Schleutker

Int J Cancer. 2018 Nov 15; 143(10): 2479–2487.  Published online 2018 Sep 22. doi: 10.1002/ijc.31746

PMCID: PMC6589920

Two genome-wide association studies of aggressive prostate cancer implicate putative prostate tumor suppressor gene DAB2IP

[David **Duggan**](https://pubmed.ncbi.nlm.nih.gov/?term=Duggan+D&cauthor_id=18073375) [^1^](https://pubmed.ncbi.nlm.nih.gov/18073375/#affiliation-1), [Siqun L Zheng](https://pubmed.ncbi.nlm.nih.gov/?term=Zheng+SL&cauthor_id=18073375), [Michele Knowlton](https://pubmed.ncbi.nlm.nih.gov/?term=Knowlton+M&cauthor_id=18073375), [Debbie Benitez](https://pubmed.ncbi.nlm.nih.gov/?term=Benitez+D&cauthor_id=18073375), [Latchezar Dimitrov](https://pubmed.ncbi.nlm.nih.gov/?term=Dimitrov+L&cauthor_id=18073375), [Fredrik Wiklund](https://pubmed.ncbi.nlm.nih.gov/?term=Wiklund+F&cauthor_id=18073375), [Christiane Robbins](https://pubmed.ncbi.nlm.nih.gov/?term=Robbins+C&cauthor_id=18073375), [Sarah D Isaacs](https://pubmed.ncbi.nlm.nih.gov/?term=Isaacs+SD&cauthor_id=18073375), [Yu Cheng](https://pubmed.ncbi.nlm.nih.gov/?term=Cheng+Y&cauthor_id=18073375), [Ge Li](https://pubmed.ncbi.nlm.nih.gov/?term=Li+G&cauthor_id=18073375), [Jielin Sun](https://pubmed.ncbi.nlm.nih.gov/?term=Sun+J&cauthor_id=18073375), [Bao-Li Chang](https://pubmed.ncbi.nlm.nih.gov/?term=Chang+BL&cauthor_id=18073375), [Leslie Marovich](https://pubmed.ncbi.nlm.nih.gov/?term=Marovich+L&cauthor_id=18073375), [Kathleen E Wiley](https://pubmed.ncbi.nlm.nih.gov/?term=Wiley+KE&cauthor_id=18073375), [Katarina Bälter](https://pubmed.ncbi.nlm.nih.gov/?term=B%C3%A4lter+K&cauthor_id=18073375), [Pär Stattin](https://pubmed.ncbi.nlm.nih.gov/?term=Stattin+P&cauthor_id=18073375), [Hans-Olov Adami](https://pubmed.ncbi.nlm.nih.gov/?term=Adami+HO&cauthor_id=18073375), [Marta Gielzak](https://pubmed.ncbi.nlm.nih.gov/?term=Gielzak+M&cauthor_id=18073375), [Guifang Yan](https://pubmed.ncbi.nlm.nih.gov/?term=Yan+G&cauthor_id=18073375), [Jurga Sauvageot](https://pubmed.ncbi.nlm.nih.gov/?term=Sauvageot+J&cauthor_id=18073375), [Wennuan Liu](https://pubmed.ncbi.nlm.nih.gov/?term=Liu+W&cauthor_id=18073375), [Jin Woo Kim](https://pubmed.ncbi.nlm.nih.gov/?term=Kim+JW&cauthor_id=18073375), [Eugene R Bleecker](https://pubmed.ncbi.nlm.nih.gov/?term=Bleecker+ER&cauthor_id=18073375), [Deborah A Meyers](https://pubmed.ncbi.nlm.nih.gov/?term=Meyers+DA&cauthor_id=18073375), [Bruce J Trock](https://pubmed.ncbi.nlm.nih.gov/?term=Trock+BJ&cauthor_id=18073375), [Alan W Partin](https://pubmed.ncbi.nlm.nih.gov/?term=Partin+AW&cauthor_id=18073375), [Patrick C Walsh](https://pubmed.ncbi.nlm.nih.gov/?term=Walsh+PC&cauthor_id=18073375), [William B Isaacs](https://pubmed.ncbi.nlm.nih.gov/?term=Isaacs+WB&cauthor_id=18073375), [Henrik Grönberg](https://pubmed.ncbi.nlm.nih.gov/?term=Gr%C3%B6nberg+H&cauthor_id=18073375), [Jianfeng Xu](https://pubmed.ncbi.nlm.nih.gov/?term=Xu+J&cauthor_id=18073375), [John D Carpten](https://pubmed.ncbi.nlm.nih.gov/?term=Carpten+JD&cauthor_id=18073375)

J Natl Cancer Inst. 2007 Dec 19;99(24):1836-44.

doi: 10.1093/jnci/djm250. Epub 2007 Dec 11. PMID: 18073375

# A meta-analysis of genome-wide association studies to identify prostate cancer susceptibility loci associated with aggressive and non-aggressive disease

[Ali Amin **Al Olama**](https://pubmed.ncbi.nlm.nih.gov/?term=Amin+Al+Olama+A&cauthor_id=23065704) [^1^](https://pubmed.ncbi.nlm.nih.gov/23065704/?utm_source=gquery&utm_medium=referral&utm_campaign=CitationSensor?otool=None#affiliation-1), [Zsofia Kote-Jarai](https://pubmed.ncbi.nlm.nih.gov/?term=Kote-Jarai+Z&cauthor_id=23065704), [Fredrick R Schumacher](https://pubmed.ncbi.nlm.nih.gov/?term=Schumacher+FR&cauthor_id=23065704), [Fredrik Wiklund](https://pubmed.ncbi.nlm.nih.gov/?term=Wiklund+F&cauthor_id=23065704), [Sonja I Berndt](https://pubmed.ncbi.nlm.nih.gov/?term=Berndt+SI&cauthor_id=23065704), [Sara Benlloch](https://pubmed.ncbi.nlm.nih.gov/?term=Benlloch+S&cauthor_id=23065704), [Graham G Giles](https://pubmed.ncbi.nlm.nih.gov/?term=Giles+GG&cauthor_id=23065704), [Gianluca Severi](https://pubmed.ncbi.nlm.nih.gov/?term=Severi+G&cauthor_id=23065704), [David E Neal](https://pubmed.ncbi.nlm.nih.gov/?term=Neal+DE&cauthor_id=23065704), [Freddie C Hamdy](https://pubmed.ncbi.nlm.nih.gov/?term=Hamdy+FC&cauthor_id=23065704), [Jenny L Donovan](https://pubmed.ncbi.nlm.nih.gov/?term=Donovan+JL&cauthor_id=23065704), [David J Hunter](https://pubmed.ncbi.nlm.nih.gov/?term=Hunter+DJ&cauthor_id=23065704), [Brian E Henderson](https://pubmed.ncbi.nlm.nih.gov/?term=Henderson+BE&cauthor_id=23065704), [Michael J Thun](https://pubmed.ncbi.nlm.nih.gov/?term=Thun+MJ&cauthor_id=23065704), [Michael Gaziano](https://pubmed.ncbi.nlm.nih.gov/?term=Gaziano+M&cauthor_id=23065704), [Edward L Giovannucci](https://pubmed.ncbi.nlm.nih.gov/?term=Giovannucci+EL&cauthor_id=23065704), [Afshan Siddiq](https://pubmed.ncbi.nlm.nih.gov/?term=Siddiq+A&cauthor_id=23065704), [Ruth C Travis](https://pubmed.ncbi.nlm.nih.gov/?term=Travis+RC&cauthor_id=23065704), [David G Cox](https://pubmed.ncbi.nlm.nih.gov/?term=Cox+DG&cauthor_id=23065704), [Federico Canzian](https://pubmed.ncbi.nlm.nih.gov/?term=Canzian+F&cauthor_id=23065704), [Elio Riboli](https://pubmed.ncbi.nlm.nih.gov/?term=Riboli+E&cauthor_id=23065704), [Timothy J Key](https://pubmed.ncbi.nlm.nih.gov/?term=Key+TJ&cauthor_id=23065704), [Gerald Andriole](https://pubmed.ncbi.nlm.nih.gov/?term=Andriole+G&cauthor_id=23065704), [Demetrius Albanes](https://pubmed.ncbi.nlm.nih.gov/?term=Albanes+D&cauthor_id=23065704), [Richard B Hayes](https://pubmed.ncbi.nlm.nih.gov/?term=Hayes+RB&cauthor_id=23065704), [Johanna Schleutker](https://pubmed.ncbi.nlm.nih.gov/?term=Schleutker+J&cauthor_id=23065704), [Anssi Auvinen](https://pubmed.ncbi.nlm.nih.gov/?term=Auvinen+A&cauthor_id=23065704), [Teuvo L J Tammela](https://pubmed.ncbi.nlm.nih.gov/?term=Tammela+TL&cauthor_id=23065704), [Maren Weischer](https://pubmed.ncbi.nlm.nih.gov/?term=Weischer+M&cauthor_id=23065704), [Janet L Stanford](https://pubmed.ncbi.nlm.nih.gov/?term=Stanford+JL&cauthor_id=23065704), [Elaine A Ostrander](https://pubmed.ncbi.nlm.nih.gov/?term=Ostrander+EA&cauthor_id=23065704), [Cezary Cybulski](https://pubmed.ncbi.nlm.nih.gov/?term=Cybulski+C&cauthor_id=23065704), [Jan Lubinski](https://pubmed.ncbi.nlm.nih.gov/?term=Lubinski+J&cauthor_id=23065704), [Stephen N Thibodeau](https://pubmed.ncbi.nlm.nih.gov/?term=Thibodeau+SN&cauthor_id=23065704), [Daniel J Schaid](https://pubmed.ncbi.nlm.nih.gov/?term=Schaid+DJ&cauthor_id=23065704), [Karina D Sorensen](https://pubmed.ncbi.nlm.nih.gov/?term=Sorensen+KD&cauthor_id=23065704), [Jyotsna Batra](https://pubmed.ncbi.nlm.nih.gov/?term=Batra+J&cauthor_id=23065704), [Judith A Clements](https://pubmed.ncbi.nlm.nih.gov/?term=Clements+JA&cauthor_id=23065704), [Suzanne Chambers](https://pubmed.ncbi.nlm.nih.gov/?term=Chambers+S&cauthor_id=23065704), [Joanne Aitken](https://pubmed.ncbi.nlm.nih.gov/?term=Aitken+J&cauthor_id=23065704), [Robert A Gardiner](https://pubmed.ncbi.nlm.nih.gov/?term=Gardiner+RA&cauthor_id=23065704), [Christiane Maier](https://pubmed.ncbi.nlm.nih.gov/?term=Maier+C&cauthor_id=23065704), [Walther Vogel](https://pubmed.ncbi.nlm.nih.gov/?term=Vogel+W&cauthor_id=23065704), [Thilo Dörk](https://pubmed.ncbi.nlm.nih.gov/?term=D%C3%B6rk+T&cauthor_id=23065704), [Hermann Brenner](https://pubmed.ncbi.nlm.nih.gov/?term=Brenner+H&cauthor_id=23065704), [Tomonori Habuchi](https://pubmed.ncbi.nlm.nih.gov/?term=Habuchi+T&cauthor_id=23065704), [Sue Ingles](https://pubmed.ncbi.nlm.nih.gov/?term=Ingles+S&cauthor_id=23065704), [Esther M John](https://pubmed.ncbi.nlm.nih.gov/?term=John+EM&cauthor_id=23065704), [Joanne L Dickinson](https://pubmed.ncbi.nlm.nih.gov/?term=Dickinson+JL&cauthor_id=23065704), [Lisa Cannon-Albright](https://pubmed.ncbi.nlm.nih.gov/?term=Cannon-Albright+L&cauthor_id=23065704), [Manuel R Teixeira](https://pubmed.ncbi.nlm.nih.gov/?term=Teixeira+MR&cauthor_id=23065704), [Radka Kaneva](https://pubmed.ncbi.nlm.nih.gov/?term=Kaneva+R&cauthor_id=23065704), [Hong-Wei Zhang](https://pubmed.ncbi.nlm.nih.gov/?term=Zhang+HW&cauthor_id=23065704), [Yong-Jie Lu](https://pubmed.ncbi.nlm.nih.gov/?term=Lu+YJ&cauthor_id=23065704), [Jong Y Park](https://pubmed.ncbi.nlm.nih.gov/?term=Park+JY&cauthor_id=23065704), [Kathleen A Cooney](https://pubmed.ncbi.nlm.nih.gov/?term=Cooney+KA&cauthor_id=23065704), [Kenneth R Muir](https://pubmed.ncbi.nlm.nih.gov/?term=Muir+KR&cauthor_id=23065704), [Daniel A Leongamornlert](https://pubmed.ncbi.nlm.nih.gov/?term=Leongamornlert+DA&cauthor_id=23065704), [Edward Saunders](https://pubmed.ncbi.nlm.nih.gov/?term=Saunders+E&cauthor_id=23065704), [Malgorzata Tymrakiewicz](https://pubmed.ncbi.nlm.nih.gov/?term=Tymrakiewicz+M&cauthor_id=23065704), [Nadiya Mahmud](https://pubmed.ncbi.nlm.nih.gov/?term=Mahmud+N&cauthor_id=23065704), [Michelle Guy](https://pubmed.ncbi.nlm.nih.gov/?term=Guy+M&cauthor_id=23065704), [Koveela Govindasami](https://pubmed.ncbi.nlm.nih.gov/?term=Govindasami+K&cauthor_id=23065704), [Lynne T O'Brien](https://pubmed.ncbi.nlm.nih.gov/?term=O%27Brien+LT&cauthor_id=23065704), [Rosemary A Wilkinson](https://pubmed.ncbi.nlm.nih.gov/?term=Wilkinson+RA&cauthor_id=23065704), [Amanda L Hall](https://pubmed.ncbi.nlm.nih.gov/?term=Hall+AL&cauthor_id=23065704), [Emma J Sawyer](https://pubmed.ncbi.nlm.nih.gov/?term=Sawyer+EJ&cauthor_id=23065704), [Tokhir Dadaev](https://pubmed.ncbi.nlm.nih.gov/?term=Dadaev+T&cauthor_id=23065704), [Jonathan Morrison](https://pubmed.ncbi.nlm.nih.gov/?term=Morrison+J&cauthor_id=23065704), [David P Dearnaley](https://pubmed.ncbi.nlm.nih.gov/?term=Dearnaley+DP&cauthor_id=23065704), [Alan Horwich](https://pubmed.ncbi.nlm.nih.gov/?term=Horwich+A&cauthor_id=23065704), [Robert A Huddart](https://pubmed.ncbi.nlm.nih.gov/?term=Huddart+RA&cauthor_id=23065704), [Vincent S Khoo](https://pubmed.ncbi.nlm.nih.gov/?term=Khoo+VS&cauthor_id=23065704), [Christopher C Parker](https://pubmed.ncbi.nlm.nih.gov/?term=Parker+CC&cauthor_id=23065704), [Nicholas Van As](https://pubmed.ncbi.nlm.nih.gov/?term=Van+As+N&cauthor_id=23065704), [Christopher J Woodhouse](https://pubmed.ncbi.nlm.nih.gov/?term=Woodhouse+CJ&cauthor_id=23065704), [Alan Thompson](https://pubmed.ncbi.nlm.nih.gov/?term=Thompson+A&cauthor_id=23065704), [Tim Dudderidge](https://pubmed.ncbi.nlm.nih.gov/?term=Dudderidge+T&cauthor_id=23065704), [Chris Ogden](https://pubmed.ncbi.nlm.nih.gov/?term=Ogden+C&cauthor_id=23065704), [Colin S Cooper](https://pubmed.ncbi.nlm.nih.gov/?term=Cooper+CS&cauthor_id=23065704), [Artitaya Lophatonanon](https://pubmed.ncbi.nlm.nih.gov/?term=Lophatonanon+A&cauthor_id=23065704), [Melissa C Southey](https://pubmed.ncbi.nlm.nih.gov/?term=Southey+MC&cauthor_id=23065704), [John L Hopper](https://pubmed.ncbi.nlm.nih.gov/?term=Hopper+JL&cauthor_id=23065704), [Dallas English](https://pubmed.ncbi.nlm.nih.gov/?term=English+D&cauthor_id=23065704), [Jarmo Virtamo](https://pubmed.ncbi.nlm.nih.gov/?term=Virtamo+J&cauthor_id=23065704), [Loic Le Marchand](https://pubmed.ncbi.nlm.nih.gov/?term=Le+Marchand+L&cauthor_id=23065704), [Daniele Campa](https://pubmed.ncbi.nlm.nih.gov/?term=Campa+D&cauthor_id=23065704), [Rudolf Kaaks](https://pubmed.ncbi.nlm.nih.gov/?term=Kaaks+R&cauthor_id=23065704), [Sara Lindstrom](https://pubmed.ncbi.nlm.nih.gov/?term=Lindstrom+S&cauthor_id=23065704), [W Ryan Diver](https://pubmed.ncbi.nlm.nih.gov/?term=Diver+WR&cauthor_id=23065704), [Susan Gapstur](https://pubmed.ncbi.nlm.nih.gov/?term=Gapstur+S&cauthor_id=23065704), [Meredith Yeager](https://pubmed.ncbi.nlm.nih.gov/?term=Yeager+M&cauthor_id=23065704), [Angela Cox](https://pubmed.ncbi.nlm.nih.gov/?term=Cox+A&cauthor_id=23065704), [Mariana C Stern](https://pubmed.ncbi.nlm.nih.gov/?term=Stern+MC&cauthor_id=23065704), [Roman Corral](https://pubmed.ncbi.nlm.nih.gov/?term=Corral+R&cauthor_id=23065704), [Markus Aly](https://pubmed.ncbi.nlm.nih.gov/?term=Aly+M&cauthor_id=23065704), [William Isaacs](https://pubmed.ncbi.nlm.nih.gov/?term=Isaacs+W&cauthor_id=23065704), [Jan Adolfsson](https://pubmed.ncbi.nlm.nih.gov/?term=Adolfsson+J&cauthor_id=23065704), [Jianfeng Xu](https://pubmed.ncbi.nlm.nih.gov/?term=Xu+J&cauthor_id=23065704), [S Lilly Zheng](https://pubmed.ncbi.nlm.nih.gov/?term=Zheng+SL&cauthor_id=23065704), [Tiina Wahlfors](https://pubmed.ncbi.nlm.nih.gov/?term=Wahlfors+T&cauthor_id=23065704), [Kimmo Taari](https://pubmed.ncbi.nlm.nih.gov/?term=Taari+K&cauthor_id=23065704), [Paula Kujala](https://pubmed.ncbi.nlm.nih.gov/?term=Kujala+P&cauthor_id=23065704), [Peter Klarskov](https://pubmed.ncbi.nlm.nih.gov/?term=Klarskov+P&cauthor_id=23065704), [Børge G Nordestgaard](https://pubmed.ncbi.nlm.nih.gov/?term=Nordestgaard+BG&cauthor_id=23065704), [M Andreas Røder](https://pubmed.ncbi.nlm.nih.gov/?term=R%C3%B8der+MA&cauthor_id=23065704), [Ruth Frikke-Schmidt](https://pubmed.ncbi.nlm.nih.gov/?term=Frikke-Schmidt+R&cauthor_id=23065704), [Stig E Bojesen](https://pubmed.ncbi.nlm.nih.gov/?term=Bojesen+SE&cauthor_id=23065704), [Liesel M FitzGerald](https://pubmed.ncbi.nlm.nih.gov/?term=FitzGerald+LM&cauthor_id=23065704), [Suzanne Kolb](https://pubmed.ncbi.nlm.nih.gov/?term=Kolb+S&cauthor_id=23065704), [Erika M Kwon](https://pubmed.ncbi.nlm.nih.gov/?term=Kwon+EM&cauthor_id=23065704), [Danielle M Karyadi](https://pubmed.ncbi.nlm.nih.gov/?term=Karyadi+DM&cauthor_id=23065704), [Torben Falck Orntoft](https://pubmed.ncbi.nlm.nih.gov/?term=Orntoft+TF&cauthor_id=23065704), [Michael Borre](https://pubmed.ncbi.nlm.nih.gov/?term=Borre+M&cauthor_id=23065704), [Antje Rinckleb](https://pubmed.ncbi.nlm.nih.gov/?term=Rinckleb+A&cauthor_id=23065704), [Manuel Luedeke](https://pubmed.ncbi.nlm.nih.gov/?term=Luedeke+M&cauthor_id=23065704), [Kathleen Herkommer](https://pubmed.ncbi.nlm.nih.gov/?term=Herkommer+K&cauthor_id=23065704), [Andreas Meyer](https://pubmed.ncbi.nlm.nih.gov/?term=Meyer+A&cauthor_id=23065704), [Jürgen Serth](https://pubmed.ncbi.nlm.nih.gov/?term=Serth+J&cauthor_id=23065704), [James R Marthick](https://pubmed.ncbi.nlm.nih.gov/?term=Marthick+JR&cauthor_id=23065704), [Briony Patterson](https://pubmed.ncbi.nlm.nih.gov/?term=Patterson+B&cauthor_id=23065704), [Dominika Wokolorczyk](https://pubmed.ncbi.nlm.nih.gov/?term=Wokolorczyk+D&cauthor_id=23065704), [Amanda Spurdle](https://pubmed.ncbi.nlm.nih.gov/?term=Spurdle+A&cauthor_id=23065704), [Felicity Lose](https://pubmed.ncbi.nlm.nih.gov/?term=Lose+F&cauthor_id=23065704), [Shannon K McDonnell](https://pubmed.ncbi.nlm.nih.gov/?term=McDonnell+SK&cauthor_id=23065704), [Amit D Joshi](https://pubmed.ncbi.nlm.nih.gov/?term=Joshi+AD&cauthor_id=23065704), [Ahva Shahabi](https://pubmed.ncbi.nlm.nih.gov/?term=Shahabi+A&cauthor_id=23065704), [Pedro Pinto](https://pubmed.ncbi.nlm.nih.gov/?term=Pinto+P&cauthor_id=23065704), [Joana Santos](https://pubmed.ncbi.nlm.nih.gov/?term=Santos+J&cauthor_id=23065704), [Ana Ray](https://pubmed.ncbi.nlm.nih.gov/?term=Ray+A&cauthor_id=23065704), [Thomas A Sellers](https://pubmed.ncbi.nlm.nih.gov/?term=Sellers+TA&cauthor_id=23065704), [Hui-Yi Lin](https://pubmed.ncbi.nlm.nih.gov/?term=Lin+HY&cauthor_id=23065704), [Robert A Stephenson](https://pubmed.ncbi.nlm.nih.gov/?term=Stephenson+RA&cauthor_id=23065704), [Craig Teerlink](https://pubmed.ncbi.nlm.nih.gov/?term=Teerlink+C&cauthor_id=23065704), [Heiko Muller](https://pubmed.ncbi.nlm.nih.gov/?term=Muller+H&cauthor_id=23065704), [Dietrich Rothenbacher](https://pubmed.ncbi.nlm.nih.gov/?term=Rothenbacher+D&cauthor_id=23065704), [Norihiko Tsuchiya](https://pubmed.ncbi.nlm.nih.gov/?term=Tsuchiya+N&cauthor_id=23065704), [Shintaro Narita](https://pubmed.ncbi.nlm.nih.gov/?term=Narita+S&cauthor_id=23065704), [Guang-Wen Cao](https://pubmed.ncbi.nlm.nih.gov/?term=Cao+GW&cauthor_id=23065704), [Chavdar Slavov](https://pubmed.ncbi.nlm.nih.gov/?term=Slavov+C&cauthor_id=23065704), [Vanio Mitev](https://pubmed.ncbi.nlm.nih.gov/?term=Mitev+V&cauthor_id=23065704), [UK Genetic Prostate Cancer Study Collaborators/British Association of Urological Surgeons' Section of Oncology](https://pubmed.ncbi.nlm.nih.gov/?term=UK+Genetic+Prostate+Cancer+Study+Collaborators%2FBritish+Association+of+Urological+Surgeons%27+Section+of+Oncology%5BCorporate+Author%5D); [UK ProtecT Study Collaborators](https://pubmed.ncbi.nlm.nih.gov/?term=UK+ProtecT+Study+Collaborators%5BCorporate+Author%5D); [Australian Prostate Cancer Bioresource](https://pubmed.ncbi.nlm.nih.gov/?term=Australian+Prostate+Cancer+Bioresource%5BCorporate+Author%5D); [PRACTICAL Consortium](https://pubmed.ncbi.nlm.nih.gov/?term=PRACTICAL+Consortium%5BCorporate+Author%5D); [Stephen Chanock](https://pubmed.ncbi.nlm.nih.gov/?term=Chanock+S&cauthor_id=23065704), [Henrik Gronberg](https://pubmed.ncbi.nlm.nih.gov/?term=Gronberg+H&cauthor_id=23065704), [Christopher A Haiman](https://pubmed.ncbi.nlm.nih.gov/?term=Haiman+CA&cauthor_id=23065704), [Peter Kraft](https://pubmed.ncbi.nlm.nih.gov/?term=Kraft+P&cauthor_id=23065704), [Douglas F Easton](https://pubmed.ncbi.nlm.nih.gov/?term=Easton+DF&cauthor_id=23065704), [Rosalind A Eeles](https://pubmed.ncbi.nlm.nih.gov/?term=Eeles+RA&cauthor_id=23065704)

Hum Mol Genet. 2013 Jan 15;22(2):408-15.

 doi: 10.1093/hmg/dds425.Epub 2012 Oct 12.

PMID: **23065704** PMCID: [PMC3526158](http://www.ncbi.nlm.nih.gov/pmc/articles/pmc3526158/)

[Replication study of 34 common SNPs associated with prostate cancer in the Romanian population](https://www.ncbi.nlm.nih.gov/pmc/articles/PMC5126261/)

Viorel **Jinga**, Irma Eva Csiki, Andrei Manolescu, Paul Iordache, Ioan Nicolae Mates, Daniel Radavoi, Stefan Rascu, Daniel Badescu, Paula Badea, Dana Mates

J Cell Mol Med. 2016 Apr; 20(4): 594–600.  Published online 2016 Jan 15. doi: 10.1111/jcmm.12729

PMCID: PMC5126261

[Genetic variations in *TERC* and *TERT* genes are associated with renal cell carcinoma risk in a Chinese Han population](https://www.ncbi.nlm.nih.gov/pmc/articles/PMC5652746/)

Dapeng **Wu**, Guodong Zhu, Jin Zeng, Wenbin Song, Ke Wang, Xinyang Wang, Peng Guo, Dalin He

Oncotarget. 2017 Sep 29; 8(44): 76832–76842.  Published online 2017 Aug 10. doi: 10.18632/oncotarget.20163

PMCID: PMC5652746

[Associations of Prostate Cancer Risk Variants with Disease Aggressiveness: Results of the NCI-SPORE Genetics Working Group Analysis of 18,343 Cases](https://www.ncbi.nlm.nih.gov/pmc/articles/PMC4586077/)

Brian T. **Helfand**, Kimberly A. Roehl, Phillip R. Cooper, Barry McGuire, Liesel M. Fitzgerald, Geraldine Cancel-Tassin, Jean-Nicolas Cornu, Scott Bauer, Erin L. Van Blarigan, Xin Chen, David Duggan, Elaine A. Ostrander, Mary Gwo-Shu, Zuo-Feng Zhang, Shen-Chih Chang, Somee Jeong, Sonja Berndt, Shannon K. McDonnell, Rick Kittles, Benjamin A. Rybicki, Matthew Freedman, Phil Kantoff, Mark Pomerantz, Joan P. Breyer, Jeffrey R. Smith, Timothy R. Rebbeck, Dan Mercola, William B Isaacs, Fredrick Wiklund, Olivier Cussenot, Stephen N. Thibodeau, Daniel J. Schaid, Lisa Cannon-Albright, Kathy A. Cooney, Stephen J. Chanock, Janet L. Stanford, June M. Chan, John Witte, Jianfeng Xu, Jeanette T. Bensen, Jack A. Taylor, William J. Catalona

Hum Genet. Author manuscript; available in PMC 2016 Apr 1.

Published in final edited form as: Hum Genet. 2015 Apr; 134(4): 439–450. Published online 2015 Feb 26. doi: 10.1007/s00439-015-1534-9

PMCID: PMC4586077

[Two Susceptibility Loci Identified for Prostate Cancer Aggressiveness](https://www.ncbi.nlm.nih.gov/pmc/articles/PMC4422072/)

Sonja I. **Berndt**, Zhaoming Wang, Meredith Yeager, Michael C. Alavanja, Demetrius Albanes, Laufey Amundadottir, Gerald Andriole, Laura Beane Freeman, Daniele Campa, Geraldine Cancel-Tassin, Federico Canzian, Jean-Nicolas Cornu, Olivier Cussenot, W. Ryan Diver, Susan M. Gapstur, Henrik Grönberg, Christopher A. Haiman, Brian Henderson, Amy Hutchinson, David J. Hunter, Timothy J. Key, Suzanne Kolb, Stella Koutros, Peter Kraft, Loic Le Marchand, Sara Lindström, Mitchell J. Machiela, Elaine A. Ostrander, Elio Riboli, Fred Schumacher, Afshan Siddiq, Janet L. Stanford, Victoria L. Stevens, Ruth C. Travis, Konstantinos K. Tsilidis, Jarmo Virtamo, Stephanie Weinstein, Fredrik Wilkund, Jianfeng Xu, S. Lilly Zheng, Kai Yu, William Wheeler, Han Zhang, African Ancestry Prostate Cancer GWAS Consortium, Joshua Sampson, Amanda Black, Kevin Jacobs, Robert N Hoover, Margaret Tucker, Stephen J. Chanock

Nat Commun. Author manuscript; available in PMC 2015 Nov 5.

Published in final edited form as: Nat Commun. 2015; 6: 6889. Published online 2015 May 5. doi: 10.1038/ncomms7889

PMCID: PMC4422072

[A novel genomic alteration of *LSAMP* associates with aggressive prostate cancer in African American men](https://www.ncbi.nlm.nih.gov/pmc/articles/PMC4703707/)

Gyorgy **Petrovics**, Hua Li, Tanja Stümpel, Shyh-Han Tan, Denise Young, Shilpa Katta, Qiyuan Li, Kai Ying, Bernward Klocke, Lakshmi Ravindranath, Indu Kohaar, Yongmei Chen, Dezső Ribli, Korbinian Grote, Hua Zou, Joseph Cheng, Clifton L. Dalgard, Shimin Zhang, István Csabai, Jacob Kagan, David Takeda, Massimo Loda, Sudhir Srivastava, Matthias Scherf, Martin Seifert, Timo Gaiser, David G. McLeod, Zoltan Szallasi, Reinhard Ebner, Thomas Werner, Isabell A. Sesterhenn, Matthew Freedman, Albert Dobi, Shiv Srivastava

EBioMedicine. 2015 Dec; 2(12): 1957–1964.  Published online 2015 Oct 31. doi: 10.1016/j.ebiom.2015.10.028

PMCID: PMC4703707

[Replication of a Genetic Variant for Prostate Cancer-Specific Mortality](https://www.ncbi.nlm.nih.gov/pmc/articles/PMC4537383/)

Kathryn L. **Penney**, Irene M. Shui, Ziding Feng, Howard D. Sesso, Meir J. Stampfer, Janet L. Stanford

Prostate Cancer Prostatic Dis. Author manuscript; available in PMC 2016 Mar 1.

Published in final edited form as: Prostate Cancer Prostatic Dis. 2015 Sep; 18(3): 260–263. Published online 2015 May 5. doi: 10.1038/pcan.2015.18

PMCID: PMC4537383

[An analysis of the association between prostate cancer risk loci, PSA levels, diseaseaggressiveness and disease-specific mortality](https://www.ncbi.nlm.nih.gov/pmc/articles/PMC4647539/)

J **Sullivan**, R Kopp, K Stratton, C Manschreck, M Corines, R Rau-Murthy, J Hayes, A Lincon, A Ashraf, T Thomas, K Schrader, D Gallagher, R Hamilton, H Scher, H Lilja, P Scardino, J Eastham, K Offit, J Vijai, R J Klein

Br J Cancer. 2015 Jun 30; 113(1): 166–172.  Published online 2015 Jun 11. doi: 10.1038/bjc.2015.199

PMCID: PMC4647539
